# Multiplex PCR Assays for the Detection of One Hundred and Thirty Seven Serogroups of Shiga Toxin-Producing *Escherichia coli* Associated With Cattle

**DOI:** 10.3389/fcimb.2020.00378

**Published:** 2020-07-29

**Authors:** Justin B. Ludwig, Xiaorong Shi, Pragathi B. Shridhar, Elisabeth L. Roberts, Chitrita DebRoy, Randy K. Phebus, Jianfa Bai, T. G. Nagaraja

**Affiliations:** ^1^Department of Diagnostic Medicine/Pathobiology, Kansas State University, Manhattan, KS, United States; ^2^E. coli Reference Center, Department of Veterinary and Biomedical Sciences, The Pennsylvania State University, University Park, PA, United States; ^3^Department of Animal Sciences and Industry/Food Science Institute, Kansas State University, Manhattan, KS, United States; ^4^Veterinary Diagnostic Laboratory, Industry/Food Science Institute, Kansas State University, Manhattan, KS, United States

**Keywords:** shiga toxin-producing *Escherichia coli* (STEC), top-7 STEC, non-top-7 STEC, Multiplex PCR assays, cattle, feces

## Abstract

*Escherichia coli* carrying prophage with genes that encode for Shiga toxins are categorized as Shiga toxin-producing *E. coli* (STEC) pathotype. Illnesses caused by STEC in humans, which are often foodborne, range from mild to bloody diarrhea with life-threatening complications of renal failure and hemolytic uremic syndrome and even death, particularly in children. As many as 158 of the total 187 serogroups of *E. coli* are known to carry Shiga toxin genes, which makes STEC a major pathotype of *E. coli*. Seven STEC serogroups, called top-7, which include O26, O45, O103, O111, O121, O145, and O157, are responsible for the majority of the STEC-associated human illnesses. The STEC serogroups, other than the top-7, called “non-top-7” have also been associated with human illnesses, more often as sporadic infections. Ruminants, particularly cattle, are principal reservoirs of STEC and harbor the organisms in the hindgut and shed in the feces, which serves as a major source of food and water contaminations. A number of studies have reported on the fecal prevalence of top-7 STEC in cattle feces. However, there is paucity of data on the prevalence of non-top-7 STEC serogroups in cattle feces, generally because of lack of validated detection methods. The objective of our study was to develop and validate 14 sets of multiplex PCR (mPCR) assays targeting serogroup-specific genes to detect 137 non-top-7 STEC serogroups previously reported to be present in cattle feces. Each assay included 7–12 serogroups and primers were designed to amplify the target genes with distinct amplicon sizes for each serogroup that can be readily identified within each assay. The assays were validated with 460 strains of known serogroups. The multiplex PCR assays designed in our study can be readily adapted by most laboratories for rapid identification of strains belonging to the non-top-7 STEC serogroups associated with cattle.

## Introduction

The polysaccharide portion, called the O-antigen, of the lipopolysaccharide layer of the outer membrane of *Escherichia coli* provides antigenic specificity and is the basis of serogrouping. As many as 187 *E. coli* serogroups have been described based on the nucleotide sequences of O-antigen gene clusters (DebRoy et al., 2016). *Escherichia coli* serogroups that cause disease in humans and animals are categorized into several pathotypes. The serogroups that carry Shiga toxin genes on a prophage are categorized as the Shiga toxin-producing *E. coli* (STEC) pathotype. As many as 158 serogroups of *E. coli* are known to carry Shiga toxin gene(s), which make STEC the most predominant *E. coli* pathotype (Table 1). Illnesses caused by STEC in humans, which are often foodborne, range from mild to bloody diarrhea with life-threatening complications of renal failure and hemolytic uremic syndrome (HUS), and even death, particularly in children (Karmali et al., 2010; Davis et al., 2014). Seven serogroups of STEC, O26, O45, O103, O111, O121, O145, and O157, called “top-7,” are responsible for the majority of human STEC illnesses, including food borne-outbreaks (Brooks et al., 2005; Scallan et al., 2011; Gould et al., 2013; Valilis et al., 2018). However, STEC serogroups other than the top-7, called “non-top-7” have also been reported to cause human illnesses, more often as sporadic infections, although a few are also known to cause severe infections, such as hemorrhagic colitis and HUS (Hussein and Bollinger, 2005; Bettelheim, 2007; Hussein, 2007; Bettelheim and Goldwater, 2014; Valilis et al., 2018). In a recent systematic review done by Valilis et al. (2018), 129 O-serogroups of STEC were identified to be associated with clinical cases of diarrhea in humans.

**Table 1:** Serogroups that belong to the Shiga toxin-producing *Escherichia coli* pathotype.

O1	O2/O50	O3	O4	O5	O6	O7	O8	O9	O10
O11/OX19	O12	O13/O129/O135	O14	O15	O16	O17/O44/O73/O77/O106	O18ab/O18ac	O19	O20/O137
O21	O22	O23	O25	O26^a^	O27	O28ac/O42	O29	O30	O32
O33	O35	O36^b^	O37	O38	O39	O40	O41	O43	O45^a^
O46/O134	O48	O49	O51	O52	O53	O54	O55	O56	O57
O58	O59	O60	O62/O68	O63	O64	O65	O66^b^	O69	O70
O71	O74	O75	O76	O78	O79	O80	O81	O82	O83
O84	O85	O86	O87	O88	O89/O101/O162	O90/O127	O91	O92	O93
O95^b^	O96	O97	O98	O100	O102	O103^a^	O104	O105	O107/O117
O108	O109	O110	O111^a^	O112	O113	O114	O115	O116	O118/O151
O119	O120	O121^a^	O123/O186	O124/O164	O125	O126	O128/OX3	O130	O131
O132	O133	O136	O138	O139	O140	O141	O142	O143	O144
O145^a^	O146	O147	O148	O149	O150	O152	O153	O154	O156
O157^a^	O158	O159	O160	O161	O163	O165	O166	O167	O168/OX6
O169	O170	O171	O172	O173	O174	O175	O176	O177	O178
O179	O180	O181	O182	O183	O184^b^	O185	O187^b^		

a *Serogroups (highlighted in blue color) considered as top-7 STEC*.

b *Serogroups (highlighted in green color) have not yet been reported in cattle feces, beef, or beef products*.

Ruminants, especially cattle, are a major reservoir of STEC and harbor the organisms in the hindgut and shed them in their feces. A number of studies have reported on the fecal prevalence of the top-7 STEC in cattle because of the availability of detection methods. For these serogroups, culture method involving serogroup-specific immunomagnetic separation and media for selective isolation and PCR assays to identify serogroups of putative isolates have been developed, validated and widely used (Bielaszewska and Karch, 2000; Chapman, 2000; Bettelheim and Beutin, 2003; Noll et al., 2015a). A number of studies have reported shedding of non-top-7 STEC in cattle feces (Table 2). However, not much is known about the prevalence of these STEC serogroups in cattle feces, in terms of their distribution and proportion of animals in a herd positive for various serogroups, largely because of lack of isolation and detection methods. Traditionally, identification of serogroups or serotyping of *E. coli*, conducted by agglutination reaction using serogroup-specific antisera, is restricted to a few reference laboratories that possess the required antisera. However, the method is time consuming and often exhibits cross-reactions with other serogroups (DebRoy et al., 2011a). A number of PCR-based assays, end point or real time, have been developed and validated for the detection of one or more clinically relevant serogroups of *E. coli* (Perelle et al., 2004; Monday et al., 2007; Fratamico et al., 2009; Bai et al., 2010, 2012; DebRoy et al., 2011b; Madic et al., 2011; Luedtke et al., 2014; Iguchi et al., 2015b; Noll et al., 2015b; Sanchez et al., 2015; Shridhar et al., 2016a). However, only a few mPCR assays have been described to detect certain STEC serogroups that are non-top-7 (Iguchi et al., 2015b; Sanchez et al., 2015; DebRoy et al., 2018).

**Table 2:** Serogroups of Shiga toxin-producing *Escherichia coli* other than the top-7 in gut contents or feces of cattle.

**Cattle type**	**Sample type**	**O-serogroups reported**	**References**
Calves with diarrhea or dysentery	Feces	O2, O5, O8, O29, O55, O149, O153	(Smith et al., 1988)
Calves	Feces	O2, O104, O128, O153	(Gonzalez and Blanco, 1989)
Bulls and dairy cows	Colonic contents of bulls at slaughter, rectal content of dairy cows	O3, O10, O22, O39, O75, O82, O91, O104, O105, O113, O116, O126, O136, O139, O156	(Montenegro et al., 1990)
Beef and dairy cattle, water buffalo	Rectal swab	O11, O25, O113, O116	(Suthienkul et al., 1990)
Dairy cattle: cows, heifers, calves; feedlot cattle	Rectal swab	O10, O15, O22, O76, O84, O116, O153, O163, O171	(Wells et al., 1991)
Dairy cows and calves	Fecal swab	O2, O3,O 4, O6, O8, O9, O11, O15, O22, O25, O32, O40, O43, O82, O87, O106, O109, O113, O117, O146, O153, O163, X3, X8	(Wilson et al., 1992)
Cattle	Rectal swab	O2, O8, O20, O22, O76, O82, O87, O88, O113, O146, O152, O156	(Beutin et al., 1993)
Cattle	Culture from cattle	O8, O9, O11, O15, O17, O20, O78, O86, O101	(Wray et al., 1993)
Dairy cows and calves	Rectal swab	O5, O18, O49, O69, O74, O76, O80, O84, O98, O118, O119, O156, O172	(Sandhu et al., 1996)
Calves, diarrheic	Feces	O4, O5, O15, O17, O53, O80, O84, O92, O118, O119, O128, O153	(Wieler et al., 1996)
Cattle	Feces	O74, O87, O90, O91, O116	(Beutin et al., 1997)
Cows and calves	Fecal swab	O2, O4, O8, O9, O20, O22, O41, O74, O77, O78, O82, O90, O91, O92, O105, O113, O116, O132, O136, O146, O150, O162, O163, O165, O171	(Blanco et al., 1997)
Calves with diarrhea	Feces	O6, O8, O25, O52, O86, O113, O167, ONT	(Beutin and Muller, 1998)
Cattle	Feces or rectal contents	O2, O16, O22, O42, O70, O74, O84, O87, O105, O109, O113, O132, O136, O146, O153, O156	(Miyao et al., 1998)
Calves, healthy and diarrheic	Feces	O118	(Wieler et al., 1998)
Dairy cows and calves	Fecal swabs	O5, O8, O22, 38, O69, O84, O98, O113, O116, O119, O132, O153, O156	(Sandhu et al., 1999)
Dairy cow with diarrhea and calves with a herd history of ill-thrift and diarrhea	Feces	O84	(Hornitzky et al., 2000)
Beef and dairy cattle: healthy and diarrheic calves; Cattle at slaughter; Grazing cows	Rectal swab	O2, O5, O20, O38, O39, O74, O79, O91, O113, O116, O117, O118, O141, O165, O168, O171	(Parma et al., 2000)
Cattle at slaughter	Feces	OX3, O1, O2, O6, O8, O15, O20, O22, O23, O39, O40, O46, O49, O74, O77, O84, O87, O88, O91, O96, O98, O102, O105, O106, O109, O112, O113, O116, O117, O120, O130, O132, O136, O140, O141, O150, O159, O163, O171, O172, OX177, OX7, OX178	(Pradel et al., 2000)
Cattle at slaughter	Rectal swab	O2, O8, O22, O43, O91, O110, O113, O116, O119, O132, O136, O153, O172	(Schurman et al., 2000)
Dairy cows and calves	Feces	O12, O35, O98, O165	(Cobbold and Desmarchelier, 2001)
Cattle	Feces	O5, O6, O7, O21, O28, O91, O113, O130, ONT	(Hornitzky et al., 2001)
Cattle at slaughter	Rectal swab	O15, O84, O91, O172	(Leung et al., 2001)
Beef and dairy cattle	Rectal swab	O20, O22, O74, O79, O84, O110, O112, O119, O125, O126, O128, O149, O156, O159, O165, O172, ONT	(Geue et al., 2002)
Beef cattle at slaughter	Fecal from cecum	O2, O8, O11, O116	(Gioffré et al., 2002)
Beef and feedlot cattle	Feces	O2, O3, O5, O6, O8, O28, O51, O68, O75, O76, O77, O81, O82, O84, O91, O93, O101, O104, O108, O110, O113, O116, O130, O149, O153, O154, O160, O163, ONT	(Hornitzky et al., 2002)
Beef or dairy cattle, calves	Feces (diagnostic samples, gastrointestinal infections)	O2, O5, O7, O8, O15, O22, O28, O41, O53, O71, O74, O75, O81, O84, O88, O98, O112, O113, O118, O119, O123, O130, O146, O159, O163, O174, O175, O177, O178, O179, O181	(Hornitzky et al., 2005)
Dairy cows, heifers, calves	Rectal swab	O29, O91, O112, O119, O125	(Moreira et al., 2003)
Cattle	Fecal swab	O22, O91, O113, O117, OX179	(Urdahl et al., 2003)
Calves	Feces	O7, O22, O113, O118, O119, O123	(Leomil et al., 2003)
Cattle, diarrheic and healthy	Feces	O2, O4, O6, O7, O8, O9, O15, O17, O20, O22, O28, O38, O39, O41, O49, O60, O64, O65, O74, O77, O79, O80, O81, O82, O84, O88, O90, O91, O96, O104, O105, O110, O113, O116, O117, O118, O123, O126, O127, O128, O132, O136, O138, O140, O141, O146, O148, O149, O150, O156, O162, O163, O165, O166, O167, O168, O171, O174, OX177, OX178, ONT	(Blanco et al., 2004a)
Cattle, grazing or feedlot	Feces	O2, O5, O8, O15, O20, O25, O38, O39, O74, O79, O91, O113, O116, O117, O118, O120, O141, O165, O168, O171, O174, O175, O177, O178, O185, ONT	(Blanco et al., 2004b)
Cattle at slaughter	Cecal content	O74, O91, O109, O110, O116, O117	(Bonardi et al., 2004)
Cattle at slaughter	Cecal content	O2, O8, O11, O25, O91, O104, O112, O113, O143, O171, O174, ONT	(Meichtri et al., 2004)
Cows and calves	Feces	O2, O8, O77, O113, O116, O136, O171, O177	(Muniesa et al., 2004)
Cattle at slaughter	Feces	O2, O5, O8, O10, O15, O35, O64, O77, O113, O119, O128, O156, O177, ONT	(Blanco et al., 2005)
Dairy cows, heifers, calves, some diarrheic	Rectal swab	O22, O44, O77, O79, O87, O88, O91, O98, O105, O112, O113, O136, O178, O181, ONT	(Irino et al., 2005)
Cattle	Feces	O2, O4, O8, O20, O22, O41, O64, O77, O82, O91, O105, O113, O116, O117, O118, O126, O128, O136, O141, O146, O150, O156, O162, O163, O168, O171, O174, O177, ONT	(Mora et al., 2005)
Cattle at slaughter	Feces	O1, O2, O5, O8, O15, O22, O86, O91, O113, O116, O117, O136, O148, O174, O182, ONT	(Zweifel et al., 2005)
Beef and dairy cattle	Feces	O2, O10, O15, O22, O74, O82, O96, O113, O116, O119, O124, O128, O137, O141, O159, O160, O63, O174, O177, O178, ONT	(Timm et al., 2007)
Steers, feedlot	Feces	O2, O8, O9, O10, O23, O37, O49, O87, O98, O132, O135, O136, O139, O153, O154, O156, O172	(Diarra et al., 2009)
Cattle	Feces	O2, O63, O148, O149, O174, ONT	(Scott et al., 2009)
Dairy cows	Feces	O2, O3, O5, O8, O11, O22, O39, O46, O64, O74, O79, O84, O88, O91, O105, O113, O130, O136, O139, O141, O163, O166, O168, O171, O1788, O179, ONT	(Fernández et al., 2010)
Beef cattle	Feces	O2, O7, O8, O15, O22, O39, O46, O73, O74, O79, O82, O91, O113, O116, O130, O136, O139, O141, O153, O163, O165, O178, O179, ONT	(Masana et al., 2011)
Cattle, beef and dairy	Pen-floor feces	O2, O13, O20, O86, O109, O113, O116, O119, O136, O168, O171, O174, ONT	(Monaghan et al., 2011)
Cattle, beef and dairy	Feces	O2, O3, O33, O69, O76, O88, O113, O118, O136, O150, O153, O171, OR, OX18	(Ennis et al., 2012)
Calves	Rectal swabs	O8, O11, O15, O91, O101, O171, ONT	(Fernández et al., 2012)
Dairy cows	Feces	O8, O21, O116, O118, O141, O153, NT	(Polifroni et al., 2012)
Beef Cattle	Rectal swabs	O2, O7, O8, O15, O22, O79, O84, O91, O107, O124, O130, O136, O141, O163, O174, O179, ONT	(Tanaro et al., 2012)
Cattle	Feces	O1, O2, O5, O8, O55, O84, O91, O109, O113, O136, O150, O156, O163, O168, O174, 177, UT	(Mekata et al., 2014)
Feedlot heifer	Colonic mucosal tissue at necropsy	O165	(Moxley et al., 2015)
Dairy Cattle	Feces	O2, O8, O10, O15, O20, O22, O39, O46, O55, O74, O77, O79, O82, O89, O91, O105, O113, O116, O141, O171, O172, O153, O165	(Gonzalez et al., 2016)
Cattle	Feces	O113, NT	(Jajarmi et al., 2017)
Cattle	Feces	O2, O3, O6, O8, O22, O28ac, O55, O71, O74, O76, O82, O87, O88, O96, O100, O104, O108, O109, O113, O115, O116, O123, O130, O132, O136, O140, O150, O153, O156, O163, O168, O171, O174, O178, O179, O183, O185	(Lee et al., 2017)
Steers	Recto anal mucosal swab	O101, O109, O177	(Stromberg et al., 2018)
Beef cattle	Feces	O178	(Paquette et al., 2018)
Dairy cattle	Feces	O3, O8, O18ac, O39, O48, O58, O77, O80, O88, O104, O112ac, O116, O146, O154, O174, O175, O176, O178, O179, O180	(Navarro et al., 2018)
Dairy cattle	Feces	O21, O22, O54, O55, O64, O69, O75, O78, O91, O92, O97, O100, O149, O173	(Peng et al., 2019)
Beef cattle	Feces	O5, O8, O15, O22, O65, O74, O76, O81, O84, O96, O116, O165, O166, O177, ONT	(Fan et al., 2019)
Beef and dairy cattle	Feces	O17, O22, O40, O76, O87, O99, O102, O108, O116, O124, O129, O136, O140, O154, O156, O163	(Bumunang et al., 2019)

*ONT, Non typeable O; UT, Untypeable; NT, nontypeable*.

In recent years, DNA microarray and whole genome sequencing have been widely used to identify *E. coli* serogroups and serotypes (Liu and Fratamico, 2006; Lacher et al., 2014; Joensen et al., 2015; Norman et al., 2015). However, mPCR assays targeting serogroup-specific genes to identify STEC is a simpler, low-cost alternative method, readily adaptable to most laboratories. Iguchi et al. (2015a) and DebRoy et al. (2016) have analyzed the nucleotide sequences of O-antigen gene clusters of 184 serogroups of *E. coli* and reported remarkable diversity among different serogroups and a high level of conservation of genes within a given serogroup in the O-antigen encoding gene clusters and suggested that these gene sequences can be targeted for serogroup identification. To understand the ecology and prevalence of these STEC serogroups in cattle, it is essential to detect the non-top-7 STEC serogroups shed in cattle feces in order to determine their impact on food safety and human health. Therefore, the objectives of the present study were to develop and validate mPCR assays targeting serogroup-specific genes to detect 137 non-top-7 STEC serogroups known to be associated with cattle.

## Materials and Methods

### Design of the Assays

A total of 14 mPCR assays, each targeting 7–12 STEC serogroups were designed. The targeted genes to design primers for serogroup detection included: *wzx*, which encodes for the O-antigen flippase required for O-polysaccharide export (Liu et al., 1996), *wzy*, which encodes for the O-antigen polymerase required for O antigen biosynthesis (Samuel and Reeves, 2003), *gnd*, which encodes for 6-phosphogluconate dehydrogenase for O antigen biosynthesis (Nasoff et al., 1984), *wzm*, which encodes for transport permease for O antigen transport, and *orf469* and *wbdC*, which encode for mannosyltransferase for O antigen biosynthesis (Kido et al., 1995). The primers were designed based on the available nucleotide sequences of the target genes for each of the STEC serogroups from the GenBank database. The sequences for each serogroup were aligned using ClustalX version 2.0. The primers were designed to amplify the target genes with distinct amplicon sizes for each serogroup within an assay for easier visualization. The forward and reverse primer sequences for these serogroups are provided in Supplementary Tables 1A–N.

### PCR Assay Conditions

The working concentrations of all primers in a primer mix were 4–7 pM/μl of each primer. The reaction consisted of 1 μL of primer mix, 10 μL of BioRad iQ Multiplex Powermix, 7 μL of sterile PCR grade water, and 2 μL of DNA template. The total reaction volume was 20 μL. The number of PCR cycles and annealing temperatures varied based on optimization for each set (Table 3). The PCR protocol for specific gene target, for sets no. 1–11, included an initial denaturation at 94°C for 5 min, followed by 25 or 30 cycles of denaturation at 94° C for 30 s, annealing for 30 s at 58–68°C, extension for 75 s at 68°C and a final step of extension at 68° C for 7 min. The assay conditions for PCR sets no. 12, 13, and 14 were initial denaturation at 94°C for 1 min, followed by 25 cycles of denaturation at 94°C for 30 s, annealing for 30 s at 58–63°C, extension for 60–80 s at 72°C and final step of extension at 72°C (Table 3). All the other conditions were similar for all 14 sets of assays. Amplicon size of PCR products was determined using a capillary electrophoresis system, QIAxcel Advanced System with QIAxcel DNA Screening Kit (Qiagen, Germantown, MD). DNA extracted from pooled strains of known serogroups for each specific set was used as positive controls and size markers for each set of assay.

**Table 3:** Multiplex PCR assays running conditions for the detection of Shiga toxin-producing *Escherichia coli* (STEC) serogroups, other than top-7 serogroups.

**Assays**	**Number of O groups**	**PCR cycles**	**Annealing temperature (^**°**^C)**	**O-serogroups (amplicon size in bp)**
Set-1	8	25	65	O109 (204), O91 (277), O168 (336), O80 (406), O156 (452), O84 (501), O86 (562), O4 (832)
Set-2	10	30	65	O5 (176), O22 (246), O171 (281), O175 (343), O13/O129/O135 (364), O119 (421), O120 (535), O123/O186 (619), O138 (696), O128 (768)
Set-3	9	30	64	O25 (230), O79 (266), O150 (313), O116 (355), O33 (413), O75 (511), O181 (595), O98 (675), O6 (783)
Set-4	10	30	63	O147 (230), O15 (288), O118/O151 (344), O113 (419), O126 (465), O178 (495), O76 (533), O146 (640), O2/O50 (819), O78 (992)
Set-5	9	30	61	O20 (204), O55 (262), O87 (306), O92 (375), O8 (448), O136 (528), O163 (596), O7 (753), O62/O68 (906)
Set-6	12	30	66	O115 (158), O39 (201), O38 (253), O74 (303), O107/O117 (357), O88 (394), O96 (457), O108 (515), O130 (567), O132 (652), O153 (741), O141 (880)
Set-7	12	30	63	O1 (152), O18ab/O18ac (199), O28 (O28ac/O42; 255), O35 (305), O37 (353), O40 (396), O43 (445), O17/O44/O73/O77/ O106 (500), O51 (566), O69 (649), O53 (735), O70 (863)
Set-8	11	25	68	O140 (155), O148 (201), O81 (248), O82 (301), O85 (353), O105 (407), O102 (453), O90/O127 (498), O124/O164 (570), O125ab/O125ac (652), O139 (859)
Set-9	9	25	63	O21 (145), O49 (197), O149 (253), O93 (299), O110 (346), O114 (396), O154 (499), O161 (646), O169 (865)
Set-10	12	25	59	O152 (150), O159 (202), O170 (233), O172 (278), O174 (317), O176 (356), O177 (395), O46/O134 (455), O179 (505), O182 (566), O160 (655), O165 (735)
Set-11	11	30	62	O3 (145), O10 (187), O11 (225), O112ab (270), O101/O162 (309), O29 (348), O23 (403), O63 (455), O16 (505), O19 (574), O131 (655)
Set-12	9	25	63	O56 (250), O9 (309), O54 (351), O27 (382), O60 (443), O143 (500), O142 (538), O48 (793), O41 (942)
Set-13	7	25	58	O133 (294), O83 (362), O167 (403), O166 (462), O64 (727), O12 (885), O58 (1046)
Set-14	8	25	58	O100 (193), O144 (245), O66 (301), O71 (344), O65 (381), O32 (452), O173 (606), O180 (744)

### Validation of PCR assays

The specificity of each assay was determined with pooled DNA of the positive controls from the other 13 sets and top-7 STEC plus O104 PCR assays. Additionally, each assay was validated with one or more strains of the targeted serogroups. A total of 460 STEC strains belonging to 137 targeted serogroups were used for the validation of the assays (Table 4; Supplementary Tables 2A–N). The strains were obtained from our culture collection (*n* = 104), *E. coli* Reference Center at Pennsylvania State University (*n* = 223), Michigan State University (*n* = 42), University of Nebraska (*n* = 5), and Food and Drug Administration (*n* = 86). Strains stored in CryoCare beads (CryoCare, Key Scientific Products, Round Rock, TX) at −80°C were streaked onto blood agar plates (Remel, Lenexa, KS) and incubated overnight at 37°C. Following incubation, colonies from the blood agar plates were suspended in 1 ml of distilled water, boiled for 10 min, centrifuged at 9,300 × g for 5 min and the supernatant was used for the PCR assays.

**Table 4:** Validation of multiplex PCR (mPCR) assays to detect “non-top-7” Shiga toxin-producing *Escherichia coli*..

**mPCR assay**	**Serogroups (No. of strains positive/No. of strains tested)**
1	O4 (5/5)^a^^,^^b^, O80 (6/6)^b^^,^^c^, O84 (4/4)^a^^,^^c^, O86 (6/6)^b^^,^^c^, O91 (4/4)^a^^,^^c^, O109 (5/5)^a^^,^^b^^,^^d^, O156 (6/6)^b^^,^^c^, O168 (5/5)^b^
2	O5 (4/4)^b^^,^^c^, O13/O129/O135 (2/2)^b^, O22 (7/7)^a^^,^^b^^,^^c^, O119 (2/2)^b^^,^^c^, O120 (5/5)^b^, O123/O186 (5/5)^b^, O128 (6/6)^b^^,^^c^, O138 (4/4)^b^^,^^c^, O171 (4/4)^a^^,^^c^, O175 (5/5)^b^
3	O6 (4/4)^a^^,^^c^, O25 (6/6)^b^, O33 (5/5)^b^, O75 (6/6)^b^^,^^c^, O79 (2/2)^b^, O98 (3/3)^a^^,^^b^, O116 (8/8)^a^^,^^c^, O150 (6/6)^b^^,^^c^, O181 (4/4)^b^
4	O2/O50 (4/4)^a^^,^^b^^,^^c^, O15 (8/8)^a^^,^^b^^,^^c^, O76 (6/6)^b^^,^^c^, O78 (4/4)^a^^,^^b^, O113 (5/5)^a^^,^^c^, O118/O151 (4/4)^a^^,^^b^^,^^c^, O126 (5/5)^a^^,^^b^^,^^c^, O146 (7/7)^a^^,^^c^, O147 (2/2)^a^^,^^c^, O178 (5/5)^b^
5	O7 (4/4)^b^^,^^c^, O8 (20/20)^a^^,^^b^^,^^c^, O20 (1/1)^a^, O55 (6/6)^a^^,^^c^, O62/O68 (4/4)^b^, O87 (3/3)^b^, O92 (1/1)^b^, O136 (6/6)^a^^,^^b^^,^^c^, O163 (4/4)^a^^,^^c^
6	O38 (3/3)^a^, O39 (3/3)^b^, O74 (4/4)^a^, O88 (5/5)^a^, O96 (4/4)^a^, O107/O117 (3/3)^a^, O108 (1/1)^a^, O115 (1/1)^b^, O130 (4/4)^a^, O132 (2/2)^a^, O141 (3/3)^b^, O153 (2/2)^a^
7	O1 (2/2)^b^^,^^e^, O17/O44/O73/O77/O106 (6/6)^b^^,^^e^, O18 (4/4)^b^^,^^e^, O28 (3/3)^b^^,^^e^, O35 (3/3)^b^^,^^e^, O37 (3/3)^b^^,^^e^, O40 (2/2)^b^, O43 (4/4)^b^^,^^e^, O51 (4/4)^b^^,^^e^, O53 (2/2)^b^^,^^e^, O69 (3/3)^b^^,^^e^, O70 (3/3)^b^^,^^e^
8	O81 (3/3)^b^^,^^e^, O82 (3/3)^b^^,^^e^, O85 (3/3)^b^^,^^e^, O90/O127 (2/2)^b^^,^^c^, O102 (4/4)^b^^,^^e^, O105 (3/3)^b^^,^^e^, O124/O164 (2/2)^b^, O125 (3/3)^b^^,^^e^, O139 (3/3)^b^^,^^e^, O140 (2/2)^b^^,^^e^, O148 (2/2)^e^
9	O21 (3/3)^b^^,^^e^, O49 (2/2)^b^^,^^e^, O93 (2/2)^b^^,^^e^, O110 (2/2)^b^^,^^e^, O114 (3/3)^b^^,^^e^, O149 (3/3)^b^^,^^e^, O154 (3/3)^b^^,^^e^, O161 (1/1)^e^, O169 (2/2)^b^^,^^e^
10	O46/O134 (5/5)^a^^,^^b^^,^^e^, O152 (2/2)^b^, O159 (2/2)^a^^,^^b^, O160 (2/2)^b^, O165 (4/4)^a^^,^^b^^,^^e^, O170 (3/3)^b^^,^^e^, O172 (2/2)^a^, O174 (2/2)^b^^,^^e^, O176 (2/2)^b^^,^^e^, O177 (5/5)^b^^,^^d^, O179 (4/4)^b^, O182 (4/4)^a^^,^^b^^,^^e^
11	O3 (3/3)^b^^,^^e^, O10 (2/2)^b^^,^^e^, O11 (3/3)^b^^,^^e^, O16 (1/1)^e^, O19 (3/3)^b^, O23 (3/3)^b^^,^^e^, O29 (3/3)^a^^,^^b^^,^^e^, O63 (3/3)^b^^,^^e^, O101/O162 (1/1)^d^, O112 (3/3)^b^^,^^e^, O131 (3/3)^b^^,^^e^
12	O9 (2)^e^, O27 (1/1)^e^, O41 (2/2)^e^, O48 (2/2)^e^, O54 (2/2)^e^, O56 (1/1)^e^, O60 (2/2)^e^, O142 (2/2)^e^, O143 (2/2)^e^
13	O12 (2/2)^e^, O58 (2/2)^e^, O64 (2/2)^e^, O83 (2/2)^e^, O133 (1/1)^e^, O166 (2/2)^e^, O167 (1/1)^e^
14	O32 (3/3)^e^, O65 (2/2)^e^, O66 (2/2)^e^, O71 (2/2)^e^, O100 (2/2)^e^, O144 (1/1)^e^, O173 (1/1)^e^, O180 (2/2)^e^

a *Strains obtained from our culture collection*.

b *Strains obtained from Pennsylvania State University*.

c *Strains obtained from Michigan State University*.

d *Strains obtained from University of Nebraska*.

e *Strains obtained from Food and Drug administration*.

## Results

**Figure 1:**
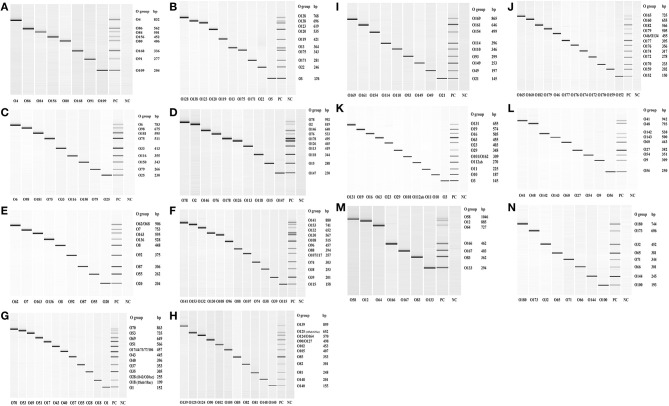
QIAxcel images of the amplicons of serogroup-specific genes of 137 serogroups of Shiga toxin-producing *Escherichia coli* amplified by 14 sets **(A–N)** of multiplex PCR assays. Positive control (PC) included pooled cultures of the known serogroups within each assay. Negative control (NC) included all reagents except the DNA template.

Out of the 158 serogroups of STEC, only five, which include O36, O66, O95, O184, and O187, have not been reported to be present in cattle feces, beef or beef products (Table 1). A total of 14 mPCR assays, each targeting 7–12 O-types of 137 non-top-7 serogroups, were designed (Table 3). Each set of mPCR assay contained primer pairs that generated amplicons of different sizes for each target serogroup that were readily differentiated using a capillary electrophoresis system (Table 3; Figures 1A–N). The PCR product size for all the assays ranged from 145 to 1,046 bp (Table 3; Figures 1A–N). The specificity of each assay was confirmed when only the genes of the targeted serogroups were amplified and none of the serogroups targeted by the other 13 sets and top-7 plus O104 PCR assays was amplified (data not shown). The assays were validated with 460 strains of known serogroups, and the results indicated that all the assays correctly identified the target serogroups (Table 4). The 14 sets of mPCR assays did not include the following 14 serogroups: O14, O30, O36, O52, O57, O59, O95, O97, O104, O158, O183, O184, O185, and O187.

## Discussion

Of the known 187 serogroups of *E. coli*, 158 serogroups have been shown to possess genes that encode for Shiga toxin 1, 2 or both. Serogroups, O26, O45, O103, O111, O121, O145, and O157, are top-7 serogroups responsible for a majority of human STEC illness outbreaks (Scallan et al., 2011; Gould et al., 2013). Among the top-7, fecal shedding of the O157 serogroup has been studied extensively, but relatively fewer studies have examined fecal shedding of the other six non-O157 serogroups in cattle, particularly in the United States (Renter et al., 2005; Cernicchiaro et al., 2013; Dargatz et al., 2013; Baltasar et al., 2014; Ekiri et al., 2014; Paddock et al., 2014; Dewsbury et al., 2015; Noll et al., 2015a; Cull et al., 2017). Among the six top-7 non-O157 serogroups, O26, O45, and O103 are the dominant serogroups in cattle feces with prevalence ranging from 40 to 50%. However, only a small proportion of these serogroups (2–6%) carry Shiga toxin genes (Noll et al., 2015a). Because Shiga toxin genes are located on a prophage, it is suggested that the serogroups lacking these genes either have lost the prophage or have the potential to acquire the prophage (Bielaszewska et al., 2007). A majority of the non-O157 top-six STEC have been show to carry Shiga toxin 1 gene (Shridhar et al., 2017). There is evidence that the type of *stx* gene carried by STEC in cattle is dependent on the age of the animal and season. Shiga toxin gene of STEC strains in adult cattle are predominantly of the *stx*2 type, whereas the strains from calves primarily possess *stx*1 type (Cho et al., 2006; Fernández et al., 2012). In a study on *E. coli* O157 in Argentina, strains of O157 detected in all seasons were predominantly of the *stx*2 type, the proportion of strains containing *stx*1 decreased and proportion of strains possessing both types increased in warm seasons (Fernández et al., 2009).

Many PCR assays have been developed and validated, generally targeting top-7 STEC serogroups, and often in combination with major virulence genes (Shiga toxins 1 and 2, intimin, and enterohemolysin: Bai et al., 2010, 2012; DebRoy et al., 2011b; Fratamico et al., 2011; Lin et al., 2011; Anklam et al., 2012; Paddock et al., 2012; Noll et al., 2015b; Shridhar et al., 2016a). There is limited development of PCR assays targeting the non-top-7 STEC in cattle feces. Individual primer pairs have been described and PCR assays have been developed for each of the 187 serogroups of *E. coli* (DebRoy et al., 2018). However, there are only a few multiplex PCR assays targeting non-top-7 STEC serogroups (Iguchi et al., 2015b; Sanchez et al., 2015). Sanchez et al. (2015) reported the development of three mPCR assays targeting 21 of the most clinically relevant STEC serogroups associated with infections in humans. The assays included, top-7 serogroups and O5, O15, O55, O76, O91, O104, O118, O113, O123, O128, O146, O165, O172, and O177. Iguchi et al. (2015b) designed primer pairs to develop 20 mPCR assays, with each set containing six to nine serogroups, to detect 147 serogroups that included STEC and non-STEC.

Because cattle are a major reservoir of STEC, we designed a series of multiplex PCR assays targeting serogroups, other than the top-7, that have been shown to be associated with feces, beef, or beef products. The nucleotide sequences of some of the targeted serogroups included in our assays have been previously shown to be 98–99.9% identical to other *E. coli* serogroups (O2/O50, O13/O129/O135, O17/O44/O73/O77/O106, O42/O28ac, O46/O134, O62/O68, O90/O127, O107/O117, O118/O151, O123/O186, O124/O164, O118/O51; DebRoy et al., 2016). Of the 158 known STEC serogroups, only five serogroups, O36, O66, O95, O184, and O187, have not been detected in cattle. The 14 sets of PCR assays did not include O104 because we have published a mPCR assay for the top-7 STEC and O104 (Paddock et al., 2013). The reason for including O104 with the top-7 STEC was because O104:H4, a hybrid pathotype of STEC and enteroaggregative *E. coli*, was involved in a major foodborne outbreak in Germany in 2011 (Bielaszewska et al., 2011). Cattle have been shown to harbor serogroup O104 in the gut and shed in the feces, however, none of the isolates was the H4 serotype and none possessed traits characteristic of the enteroaggregative *E. coli* (Paddock et al., 2013; Shridhar et al., 2016b). The 14 sets of mPCR assays did not include the following 13 serogroups: O14, O30, O36, O52, O57, O59, O95, O97, O158, O183, O184, O185, and O187. Of the 13 serogroups, O14 and O57 have been shown to contain no O-antigen biosynthesis gene clusters (Iguchi et al., 2015a; DebRoy et al., 2016). The reason for not including the remaining 11 serogroups (O30, O36, O52, O59, O95, O97, O158, O183, O184, O185, and O187) was because we were unable to procure known strains of the serogroups required for validation.

STEC serogroups other than the top-7 have been reported to be involved in sporadic cases and a few outbreaks of human illness (McLean et al., 2005; Espie et al., 2006; Buchholz et al., 2011; Mingle et al., 2012). Among the non-top-7 STEC, certain serogroups, such as O1, O2, O8, O15, O25, O43, O75, O76, O86, O91, O101, O102, O113, O116, O156, O160, and O165, specifically certain serotypes within these serogroups, have been involved in outbreaks associated with consumption of contaminated beef in the US and European countries (Eklund et al., 2001; Hussein, 2007). Many of the outbreaks included cases of hemorrhagic colitis and HUS. Serogroups O91 (mostly H21 and H14 serotypes) and O113 (mostly H21 serotype) have been associated with severe cases of hemorrhagic colitis and HUS in the US and other countries (Feng et al., 2014, 2017). Obviously, the difference in virulence between serogroups and serotypes is attributable to specific virulence factors encoded by genes in the chromosome, particularly on large horizontally acquired pathogenicity islands, or on plasmids (Levine, 1987; Bolton, 2011).

In contrast to humans, cattle are generally considered to be not susceptible to STEC infections. Only new born calves, particularly those that are immunocompromised because of deprived colostrum, have been shown to exhibit *E. coli* O157:H7 infections characterized by bloody diarrhea and attaching and effacing lesions (Dean-Nystrom et al., 1998; Moxley and Smith, 2010). Other serogroups that have been associated with diarrheal diseases of calves include O5, O8, O20, O26, O111, and O113 (Mainil and Daube, 2005). The majority of the serotypes causing infections in calves carried only Shiga toxin 1 gene (Mainil and Daube, 2005). Moxley et al. (2015) have reported isolation of STEC O165:H25 from the colonic mucosal tissue of an adult heifer that died of hemorrhagic colitis.

Some of the serogroups detected in cattle feces such as O5, O8, O9, O11, O15, O20, O49, O59, O62, O65, O69, O71, O76, O78, O86, O87, O89, O91, O100, O114, O115, O116, O119, O120, O128, O138, O139, O141, O143, O147, O159, O163, O167, O172, O174, and O180 have also been detected in swine feces (Cha et al., 2018; Peng et al., 2019). A few of the swine STEC serogroups, particularly O8, O138, O139, O141, and O147, are more often implicated in edema disease in weaned piglets and young finishing pigs (Kaper et al., 2004; Melton-Celsa et al., 2012).

Of the 158 STEC serogroups, 130 serogroups have been associated with clinical cases of diarrhea in humans (Mainil and Daube, 2005; Hussein, 2007; Valilis et al., 2018). Therefore, there are 28 STEC serogoups that have not been reported to cause human infections, which is interesting because Shiga toxins are potent virulence factors. Either these STEC have not yet been linked to an illness or they lack other virulence factors, such as those needed for attachment and colonization, necessary to cause infections. A further understanding and assessment of the virulence potential of these serogroups will require sequencing of the whole genome to obtain a comprehensive gene profile.

In conclusion, the multiplex PCR assays designed in our study, which can be readily performed in most microbiology laboratories, will allow for rapid identification of isolates belonging to the non-top-7 *E. coli* STEC serogroups that are prevalent in cattle feces, beef or beef products.

## Data Availability Statement

The raw data supporting the conclusions of this article will be made available by the authors, without undue reservation.

## Author Contributions

JB, TN, and CD conceived and designed the experiments. JL and XS performed the experiments. XS, JB, CD, ER, RP, and TN contributed reagents, materials, and analysis tools. PS, CD, XS, JB, and TN wrote the paper. All authors contributed to the article and approved the submitted version.

## Conflict of Interest

The authors declare that the research was conducted in the absence of any commercial or financial relationships that could be construed as a potential conflict of interest.
