# Supplementary material for: Multiplex PCR Assays for the Detection of One Hundred and Thirty Seven Serogroups of Shiga Toxin-Producing Escherichia coli Associated With Cattle
Source: Front Cell Infect Microbiol. 2020 Jul 29;10:378. doi: 10.3389/fcimb.2020.00378 (PMC7403468; doi:10.3389/fcimb.2020.00378)
Supplement: Supplementary file 1 [file Data_Sheet_1.PDF]

Table 1A. Target serogroups, serogroup-specific genes, primers and amplicon size of multiplex PCR assay set no. 1 for the detection of non-top 7 STEC serogroups

Serogroups	Target genes	Primers	Amplicon size (bp)	Reference
O4	*wzx*	F: TGAAACAGCAGTGCGTGCATTCTC<br>R: GATTGCCGTGCCAATTATGCCGAA	832	DebRoy et al., 2018
O80	*wzx*	F: GAAGCATGGCTTCTAGGGGG<br>R: TGACAAAGGTAGCCACGGAA	406	DebRoy et al., 2018
O84	*wzx*	F: TCAGCGTTCCAAGAAGCACT<br>R: TGGTGTGCACTTATACATCCGA	501	DebRoy et al., 2018
O86	*wzy*	F: ATTTGAGGCTGACGCGTATGGACT<br>R: AGCAACACTTCCAATGATCCACCC	562	This study
O91	*wzy*	F: CGCATTTAAGGACTGGCTGT<br>R: GTAGCAGATATGCCGACCGT	277	Fratamico et al., 2009
O109	*wzx*	F: TCTCTCTCGACATACCCGCGCTT<br>R: ACCGTAGCCCAAAGAGCCACA	204	Iguchi et al., 2015
O156	*wzx*	F: TGCTCATGCGTTTAAAATGG<br>R: CAACAAGAACAGGCATCAGG	452	This study
O168	*wzx*	F: TGTCGACTTTGGGAAATGTGG<br>R: CTGCAGAGGCCAATTCAGGT	336	DebRoy et al., 2018

Table 1B. Target serogroups, serogroup-specific genes, primers and amplicon size of multiplex PCR assay set no. 2 for the detection of non-top-7 STEC serogroups

Serogroups	Target genes	Primers	Amplicon size (bp)	Reference
O5	*wzy*	F: AGCCCAAGAGGCCGCAGTATTT<br>R: TCCAACCACCATTCTCCGCCAT	176	This study
O13/O129/<br>O135	*wzx*	F: CGGGAGAGCAGTGTTTCCAA<br>R: TATTGCGGCACCCAGTAACC	364	DebRoy et al., 2018
O22	*wzy*	F: TTTACTGGCTGCTGCTAGTGCT<br>R: TCATCTCCACCACGAGTCGAAAGT	246	Fratamico et al., 2009
O119	*wzx*	F: CTGGGGCAATCTGCTTTCCT<br>R: CCCAAGGTATTGTTGCCCCT	421	DebRoy et al., 2018
				DebRoy et al., 2018
O120	*wzx*	F: CTGGTTTTGTTGTTGCATTGCT<br>R: CCAGTTGGTGCCAACCAAAG	535	DebRoy et al., 2018
				DebRoy et al., 2018
O123/O186	*wzx*	F: ACAATTAGGGCCTGGTGCAT<br>R: TGTGCTAGCGCTAAAGGACT	619	DebRoy et al., 2018
				DebRoy et al., 2018
O128	*wzx*	F: GCCATTACGACGTTGATGACT<br>R: TGCAACCCCAATAGCAAAAGC	768	DebRoy et al., 2018
				DebRoy et al., 2018
O138	*wzx*	F: GCAGCAATGCCTGCTGTTTT<br>R: AGCGTATGCAACCCCAATGA	696	DebRoy et al., 2018
O171	*wzx*	F: TGCTCAAGTGGCATGCAGAT<br>R: TGCAACCTGATATCCAGCAGT	281	DebRoy *et al.*, 2018
O175	*wzx*	F: TGTTTAATTGTTCCTCCGCTCA<br>R: GCAGCCCAACCAAACTTAGC	343	DebRoy *et al.*, 2018

Table 1C. Target serogroups, serogroup-specific genes, primers and amplicon size of multiplex PCR assay set no. 3 for the detection of non-top 7 STEC serogroups

Serogroups	Target genes	Primers	Amplicon size (bp)	Reference
O6	*wzy*	F: GGATGACGATGTGATTTTGGCTAAC<br>R: TCTGGGTTTGCTGTGTATGAGGC	783	Grozdanov et al., 2002
O25	*wzy*	F: AGAGATCCGTCTTTTATTTGTTCGC<br>R: GTTCTGGATACCTAACGCAATACCC	230	Li et al., 2010
O33	*wzx*	F: GGACCTCTGTTTGTGGCTGT<br>R: GGTGTGCCTATCGCATAACC	413	DebRoy et al., 2018
O75	*wzy*	F: GAGATATACATGGGGAGGTAGGCT<br>R: ACCCGATAATCATATTCTTCCCAAC	511	Li et al., 2010
O79	*wzx*	F: GCTATAGTGCACCAGGATTGT<br>R: GATTGCCGTCTGCCCTAAT	266	DebRoy et al., 2018
O98	*wzx*	F: GGGCGTATTGAGGTTCTTGT<br>R: CAACTGAAATGGAGCAGCAAAT	675	DebRoy et al., 2018
O116	*wzx*	F: CTTTTGCGTTGTGCCTCGAA<br>R: ATACTGCCCTACGTTTGCGG	355	DebRoy et al., 2018
O150	*wzx*	F: TGCGTTCACTTGCTGGTTTG<br>R: ATGAGTGCAGGCACTTGAA	313	DebRoy et al., 2018
O181	*wzx*	F: TGGAGTAACGAAATACACCGCT<br>R: AGATTGCCAATAACCAGAAGCA	595	DebRoy et al., 2018

Table 1D. Target serogroups, serogroup-specific genes, primers and amplicon size of multiplex PCR assay set no. 4 for the detection of non-top 7 STEC serogroups

Serogroups	Target genes	Primers	Amplicon size (bp)	Reference
O2/O50	*wzx*	F: TGGCCTTGTTCGATATACTGCGGA<br>R: TCACGAGCTGAGCGAAACTGTTCA	819	Fratamico et al., 2010
O15	*wzx*	F: CCAAATTGGTTTTTGCAAGG<br>R: TGCACTAGTCGCAAGTGTTGA	288	This study
O76	*wzx*	F: CATATGCAGATTGAAGGTAG<br>R: GAAAGCCATAAAGTGCC	533	DebRoy et al., 2018
O78	*wzx*	F: GGTATGGGTTTGGTGGTA<br>R: AGAATCACAACTCTCGGCA	992	Liu et al., 2010
O113	*wzy*	F: GCATGTATGATGCATAGCTTCGCC<br>R: TGATATCGTTCGCTAACCACCCA	419	DebRoy et al., 2004
O118/O151	*wzx*	F: GTGGGAGTCTGAATCAAGTTGCGA<br>R: AGCAACCTTACCCAATCCTAAGGG	344	Liu et al., 2008
O126	*wzy*	F: CGCATTAAATGGACCTGATAAAGCATCG<br>R: ACTAGCGCACATATCGTTAGCACG	465	Liu et al., 2007
O146	*wzx*	F: AGGGTGACCATCAACACACTTGGA<br>R: AGTTCAATACTGTCGCAGCTCCTC	640	Liu et al., 2007
O147	*wzx*	F: GCTTGATTGGCGGTAGTGTAT<br>R: TGGTAATCCAGCCACAAAAGAA	230	This study
O178	*wzx*	F: CTGTCCGTAGTGAGGTTGGC<br>R: ACCTCCAGATCGGTCCTTAATC	495	DebRoy et al., 2018

Table 1E. Target serogroups, serogroup-specific genes, primers and amplicon size of multiplex PCR assay set no. 5 for the detection of non-top 7 STEC serogroups

Serogroups	Target genes	Primers	Amplicon size (bp)	Reference
O7	*wzx*	F: GCCAGGCAAGGATTATTATGG<br>R: TGTCATTGCAGGTACGCTAGA	753	This study
O8	*Orf469*	F: CCAGAGGCATAATCAGAAATAACAG<br>R: GCAGAGTTAGTCAACAAAAGGTCAG	448	Li et al., 2010
O20/O137	*wzx*	F: GAGCAGCGGAATACTTTCCA<br>R: TCCAAGAGTCCTGATGCAAAT	204	This study
O55	*wzx*	F: TGCCAATACGTAATACCCAAAA<br>R: GTGGCCACAGGCAATCTTAT	262	This study
O62/O68	*wzx*	F: TGAAATGTTTGAGCGTGAATTT<br>R: CATATCGCCGAATGAGTAACC	906	This study
O87	*wzx*	F: TGGGTTTTTGTCAAGCATCA<br>R: TCGCTTTCATTTTCTCCATTC	306	This study
O92	*gnd*	F: TGCGAAGTGGTCTGAATCTG<br>R: TTCCCAAGTACAATGCACGA	375	This study
O136	*wzx*	F: GCCACGTACAAAAATTTAGCC<br>R: CCCCTTCTTGATCCATTTGA	528	This study
O163	*wzy*	F: TTGCTGATGATGAGGTTGTGA<br>R: ATGTTCCACTCCCTGCAAGT	596	This study

Table 1F. Target serogroups, serogroup-specific genes, primers and amplicon size of multiplex PCR assay set no. 6 for the detection of non-top 7 STEC serogroups

Serogroups	Target genes	Primers	Amplicon size (bp)	Reference
O38	*wzx*	F: AAGAGGCTGGGGCATTTAGT<br>R: TGCGCCGATAAATCCATATT	253	This study
O39	*wzx*	F: CACAAGCATTTCAGCCAAAG<br>R: TGAGAAAATCACCAGCCTGC	201	This study
O74	*wzx*	F: CTGGTCAATGGCAAGCTGTA<br>R: ATGCAAAAATCCAAGCCAAT	303	This study
O88	*wzx*	F: TTCTCTCCCTTCGTTGGCTA<br>R: TTCCCACACCAGCATTAACA	394	This study
O96	*wzy*	F: GGGCTGTTTACGAGAGCATT<br>R: GCACTACTAGGATCATCCGCA	457	This study
O107/O117	*wzx*	F: TTTGATGTCGTTGCTCCGTA<br>R: TGCACCAATTGTCGGTGA	357	This study
O108	*wzx*	F: GGTGGTCGTGAGCAACTAGG<br>R: TTATACGCGAGATGCTTTGC	515	This study
O115	*wzx*	F: GTGAATTTGCTCGCCTCTCT<br>R: TGCTACCAACCATTTATGCG	158	This study
O130	*wzx*	F: GAGGGCTAATTGCATCCGTA<br>R: GCAACAAATGAACGCATGAC	567	This study
O132	*wzx*	F: TGGCAATCCCTCTGATCTTC<br>R: ATCAAACATAACCCGCCTGA	652	This study
O141	*wzx*	F: GTGTATTTAACATTTGCTCAAGCC<br>R: ACGCTGAAGGAATCGTCAAC	880	This study
O153	*wzx*	F: TTCGTCCTATGCGCAGTATTC<br>R: ATTGCCTGAACACGGAAGAT	741	This study

Table 1G. Target serogroups, serogroup-specific genes, primers and amplicon size of multiplex PCR assay set no. 7 for the detection of non-top 7 STEC serogroups

Serogroups	Target genes	Primers	Amplicon size (bp)	Reference
O1	*wzx*	F: CTTTTAGTGCAAGTCGTGCG<br>R: AGCTTGCACAGGCAAAAGAT	152	This study
O18	*wzx*	F: CGAGAAGTCGCAATTGAAAA<br>R: TGGATGCTGCGAGAATTTTA	199	This study
O28	*wzx*	F: CTCAAGTCATTGGCGCATTA<br>R: GGCTGACTGGGGTCGTTAT	255	This study
O35	*wzx*	F: TGGCATAGCGGTGTTCTATG<br>R: CCCAAGAAAGTGGAAAAACAA	305	This study
O37	*wzx*	F: TAGCCATATGCCTTGTCGTG<br>R: TGAATCGCTGGTAACGAACA	353	This study
O40	*wzx*	F: CCTGAAGAGGCTGGGAGAAT<br>R: AAGAAAACTCATTGGCACACC	396	This study
O43	*wzx*	F: TTGAATCGAAGGCTTTTTGC<br>R: AGGTATCACACCATGCACGA	445	This study
O44/17/73/7<br>7/106	*wzx*	F: GGAGAAGATGTAGGCGGAGA<br>R: AGCACATCAAAAGCTGACGTT	500	This study
O51	*wzy*	F: TTGGTCGCCTTATTTATTGCTT<br>R: AACGTAATGAAAAACCAGGAACA	566	This study
O53	*wzx*	F: CGACCTCCTTTATTGGTTGC<br>R: TCTTTCCCATAATTCAATCCAA	735	This study
O69	*wzx*	F: ATCATTTGCAGGGCTTATGG<br>R: CTTCCTTTCATCCCGAGAAT	649	This study
O70	*wzx*	F: ATACCGGTCCATCAGGAATG<br>R: AACATCCCGCGAAAAATAAA	863	This study

Table 1H. Target serogroups, serogroup-specific genes, primers and amplicon size of multiplex PCR assay set no. 8 for the detection of non-top 7 STEC serogroups

Serogroups	Target genes	Primers	Amplicon size (bp)	Reference
O81	*wzx*	F: AGATCGCAGGCTTCGTTG<br>R: GGGCAGGACCTTCTTAAACA	248	This study
O82	*wzx*	F: TCGGGATATCTGGATTTGGA<br>R: AGCCAATTCATTTGGAATGTTT	301	This study
O85	*wzx*	F: TGGATACTGCAGGTGAGTGG<br>R: AATGCTAAGTACAGCGCAACC	353	This study
O90/O127	*wzx*	F: CAGTTCGGCTGGTTGGTT<br>R: CCCAGTTGTGCAACAATAACA	498	This study
O102	*wzx*	F: GCTCGTGAATTGGGAGCA<br>R: TGCACATGTCAGGGTTGG	453	This study
O105	*wzx*	F: TCAAAGATGAGAGTGGGAATAGC<br>R: CGGCTCTTCATCATTGCAT	407	This study
O124/O164	*wzx*	F: CCGCGATGAATGATTCTGTA<br>R: TCACTCCCTGAACTCTGCAC	570	This study
O125	*wzx*	F: GAGAGGAGCATTGATTGCTGT<br>R: TTGAGCATTTTCGACGTAGC	652	This study
O139	*wzx*	F: GGGATTAGGCGGCTTTTTAAT<br>R: TACTTGCCCCAAAAGAGACG	859	This study
O140	*wzx*	F: AAACGCAATAAGTGGGTTGG<br>R: GCAATTGGACCAGAAACAAA	155	This study
O148	*wzx*	F: TCGCTAAACTGACAGGTGTGAT<br>R: ACGGCCACGCTTCTTTTA	201	This study

Table 1I. Target serogroups, serogroup-specific genes, primers and amplicon size of multiplex PCR assay set no. 9 for the detection of non-top 7 STEC serogroups

Serogroups	Target genes	Primers	Amplicon size (bp)	Reference
O21	*wzx*	F: GCTCAATATGAGTGAGGCTGGT<br>R: AAAAACGCCTCGTGCTTTTA	145	This study
O49	*wzx*	F: TTTACCGCGAATAACGGTTC<br>R: ACAGGGGCAATACGTTCATC	197	This study
O93	*wzx*	F: GGCAGGAGAATATGCAAGGA<br>R: TAAATGCCTCCCAAACACCA	299	This study
O110	*wzx*	F: TGTTGCAAGCATGAAACTTGA<br>R: TAAGCCATTTTCGCCAAATC	346	This study
O114	*wzx*	F: TGCTACAAGTGGTGGATTGC<br>R: CCCATACAACCATCGCAAAT	396	This study
O149	*wzx*	F: AGCGGTGCAAAGTTAATTCC<br>R: CACCAAACACATTCTGCGTAA	253	This study
O154	*wzx*	F: TTGGTTTTTCCAAGGGATACA<br>R: CAAAGCAAATACCACCATCG	499	This study
O161	*wzx*	F: GGATATCTGGTTGGCGACAT<br>R: AGCTTAAAACATGAGGGAGCTG	646	This study
O169	*wzx*	F: GAAGGAATGGGATTTGAAAAGA<br>R: CGCTTTAACAATTGCTTTCG	865	This study

Table 1J. Target serogroups, serogroup-specific genes, primers and amplicon size of multiplex PCR assay set no. 10 for the detection of non-top 7 STEC serogroups

Serogroups	Target genes	Primers	Amplicon size (bp)	Reference
O46/O134	*wzx*	F: TCAGGTGCGCCATTATTTTT<br>R: TCACCCCCATAATAACCATCTT	455	This study
O152	*wzx*	F: TCTGTCGCTATTGTCTTTTCACA<br>R: TGCCGTTAACGTACCAAAAA	150	This study
O159	*wzx*	F: AAATCCATTGGTGGAGTAGGAA<br>R: CTTCCGCAAATAAGGTCCAA	202	This study
O160	*wzx*	F: TGGCGTTCTAGGTCTTGCTAT<br>R: TGCAGCCCCTCTGATTCTAT	655	This study
O165	*wzx*	F: TGCGATTGCTTTATTTGAGC<br>R: TCACGCTTTAACGCATACAGA	735	This study
O170	*wzx*	F: AATAGAATCGCCTGGGGATAG<br>R: CAGTGCTTAAAGGATGGTTGG	233	This study
O172	*wzx*	F: CGGGCATGTTTGTTTTTGAT<br>R: AGCATCCCACCTCTCACAAT	278	This study
O174	*wzx*	F: GGAGATAAAGCTGCAGGTGAG<br>R: CCAGTAAGCGGGCCTAAAAG	317	This study
O176	*wzx*	F: TTTTTAGATTTGTTGATCCATTGGT<br>R: CATAAGCGGCACTGCAATTA	356	This study
O177	*wzx*	F: GGGGTATACACGCTCGCTAT<br>R: CTTACAGCAGGCAACGCAAC	395	This study
O179	*wzx*	F: ATGCCATTGCCGATGATATG<br>R: ACCTCCACCCCAGCATAACT	505	This study
O182	*wzx*	F: TTAGTTAATGCAGCCGTCACA<br>R: GCCGATACTCCCATAAAAAGC	566	This study

Table 1K. Target serogroups, serogroup-specific genes, primers and amplicon size of multiplex PCR assay set no. 11 for the detection of non-top 7 STEC serogroups

Serogroups	Target genes	Primers	Amplicon size (bp)	Reference
O3	*wzx*	F: CGCTTAATTCGACCCAAATC<br>R: AGTCGTTCTTGAGCCATCTGTA	145	This study
O10	*wzx*	F: AGGAAAACATTTGCCGGAAT<br>R: TATGGAGCGGTGATGAAACA	187	This study
O11	*wzx*	F: CCATTGGTCACACTGCCTTA<br>R: CAAACCAAGAGATGCTGCAA	225	This study
O16	*wzx*	F: TTTTGGGCGGTGATATTTTC<br>R: CCGCATTAAAATTCCCTACG	505	This study
O19	*wzx*	F: GCGGAATATCTCGTGCAGTT<br>R: GCGAAATGTGAGACAGCAAA	574	This study
O23	*wzx*	F: TTTATCTTGGGCGGCTTAAA<br>R: CCCACCCAAGCATTTACTTT	403	This study
O29	*wzx*	F: TTATTGGGTTGTCGCCATTT<br>R: CGGTCAACTTGTGAAAGCAA	348	This study
O63	*wzx*	F: GGGCAGATGCAAGGATTTAC<br>R: AAGACTCCTCCCCCAATCAG	455	This study
O101/O162/O89	*wzm*	F: TGGACCACATTAGGTTGGTTAGAG<br>R: AAGCATCATAAAATTTCGCCATAG	309	This study
O112ab	*wzx*	F: ACAACGTGAAATTTTTGTGTGG<br>R: GCACGTATGCATTCTCAAGC	270	This study
O131	*wzx*	F: GTGATTTCTGGGGCAACATT<br>R: AAGCCTGCCCTAAACAAAGC	655	This study

Table 1L. Target serogroups, serogroup-specific genes, primers and amplicon size of multiplex PCR assay set no. 12 for the detection of non-top 7 STEC serogroups

Serogroups	Target genes	Primers	Amplicon size (bp)	Reference
O9	*wbdC*	F: GCAGGAGCGATTCCTCG<br>R: CGCCGCACTTCCGCC	309	This study
O27	*wzy*	F: AACCCTATGGGAAGCTCTGGA<br>R: ACACACAGGCAACAACATCGA	382	Iguchi et al., 2015
O41	*wzx*	F: TGGATCGCTCGTTATTTGG<br>R: CGCCACCCCTTGGTATATAAA	942	Iguchi et al., 2015
O48	*wzy*	F: TATGGTGCTGCTTTCTCCAA<br>R: AGGAATTGCAGTTGTTCCGA	793	Iguchi et al., 2015
O54	*wzy*	F: TGGCAATATATGCGTTTGTGA<br>TGTGGACCACGTCCAACTTC	351	Iguchi et al., 2015
O56	*wzx*	F: CTTGGGGTTTGAAGGTTGGAT<br>R: TGCTAATAACAATGCGCCTG	250	Iguchi et al., 2015
O60	*wzm*	F: TAGGTGCGGCATGGCTAATAT<br>R: GAATTGGCCAACATCACGAA	443	Iguchi et al., 2015
O142	*wzy*	F: TGGGCCTGCATCATTTTTC<br>R: GGGCACGTTGACGTAATCTAA	538	Iguchi et al., 2015
O143	*wzy*	F: TGGCCTGCATGCTCTTTTT<br>R: ATATACCCCTCCGAGGACAAA	500	Iguchi et al., 2015

Table 1M. Target serogroups, serogroup-specific genes, primers and amplicon size of multiplex PCR assay set no. 13 for the detection of non-top 7 STEC serogroups

Serogroups	Target genes	Primers	Amplicon size (bp)	Reference
O12	*wzy*	F: CAATGGGGTTGTCGTATCAAA<br>R: AAAAATGCCCCATAGGACCA	885	Iguchi et al., 2015
O58	*wzy*	F: TAGGTGCAAGTCCTATGTGGG<br>R: TAGCCTGGCAGCACAGAGTTT	1046	Iguchi et al., 2015
O64	*wzy*	F: TGGGCAATACAAGTCTGATGC<br>R: AGGGCGTTACCGGATAGAAAT	727	Iguchi et al., 2015
O83	*wzx*	F: GTACACCAGGCAAACCTCGAAAG<br>R: TTCTGTAAGCTAATGAATAGGCACC	362	Li et al., 2010
O133	*wzx*	F: TAAAGGCTGCTGCTCAAGGT<br>R: GCCCTTGCGTCAAGTATAGG	294	This study
O166	*wzy*	F: TTCATAGCTGGCCTCCTTGTT<br>R: TCTATTCGCCGAATCCTTTCT	462	Iguchi et al., 2015
O167	*wzy*	F: TCAGGGGCAATTACAATCCTT<br>R: TCGCGCATAGAATAGCATGTC	403	Iguchi et al., 2015

Table 1N. Target serogroups, serogroup-specific genes, primers and amplicon size of multiplex PCR assay set no. 14 for the detection of non-top 7 STEC serogroups

Serogroups	Target genes	Primers	Amplicon size (bp)	Reference
O32	*wzy*	F: TCCCAACCCTGTTGCTTTAA<br>R: CAGCCAGACCAGTAGAGGAAA	452	Iguchi et al., 2015
O65	*wzy*	F: TGTTGGCGCTGGTTTTATGTT<br>R: CCCATAATTGCACCGCATAA	381	Iguchi et al., 2015
O66	*wzy*	F: CGAGCAAATTAAATCCAC<br>R: TCAACACTAAACGAAACG	301	Cheng et al., 2007
O71	*wzx*	F: GCATTATTAGCCACTTCAA<br>R: AGCCGTATCATTTAGAGCAGA	344	Hu et al., 2010
O100	*wzx*	F: TGCAACGATTATTGGTGTCG<br>R: ATACAAACCCGCTTGAACCA	193	This study
O144	*wzx*	F: TGCGAGATTCAGCATATTGG<br>R: CAACCCCTCCCCATAAACTC	245	This study
O173	*wzy*	F: TTCAAAGTGCTCTGGAGGGA<br>R: TGGCTGAGACTTGACTATTTT	606	Wang et al., 2010
O180	*wzy*	F: TGGCATCAACGAATGATGCA<br>R: TTGCCCATGCTTCACCAATA	744	Iguchi et al., 2015
