# Supplementary material for: Multiplex PCR Assays for the Detection of One Hundred and Thirty Seven Serogroups of Shiga Toxin-Producing Escherichia coli Associated With Cattle
Source: Front Cell Infect Microbiol. 2020 Jul 29;10:378. doi: 10.3389/fcimb.2020.00378 (PMC7403468; doi:10.3389/fcimb.2020.00378)
Supplement: Supplementary file 2 [file Data_Sheet_2.PDF]

Table 2A. Validation of multiplex PCR assay set no. 1 to detect 'non-top-7' Shiga toxin producing *Escherichia coli* serogroups

Serogroups	Source of strains, Strain IDs					No. of strains positive/No. of strains tested
	Kansas State University	Pennsylvania State University	Michigan State University	University of Nebraska	Food and Drug Administration	
O4	6282	O4 std., 14.0484, 14.0486, 14.1232				5/5
O80		O80 std., 13.0406, 12.3395, 9.1802 9.1803				5/5
O84	10340-1, 12824-1					2/2
O86		O86 std., 14.0607, 14.0615, 14.0747, 14.0748				5/5
O91	4162, sPRH-561					2/2
O109	12662-2, 15150-1, 15166-1	13.0166		E10F-6		5/5
O156						4/4

	O156 std., 15.0353, 15.0354, 15.0355		
O168	O168 std., 15.0133, 15.0181, 15.0185, 14.0047		5/5

Table 2B. Validation of multiplex PCR assay set no. 2 to detect 'non-top-7' Shiga toxin producing *Escherichia coli* serogroups

Serogroups	Source of strains, Strain IDs					No. of strains positive/No. of strains tested
	Kansas State University	Pennsylvania State University	Michigan State University	University of Nebraska	Food and Drug Administration	
O5		14.0590, 14.1660				2/2
O13/O129/O135		13.0102, 12.2598				2/2
O22	1245, 4590, 4570, 1027-4	13.0145				5/5
O119		15.1553				1/1
O120		O120 std., 13.0021, 13.0022, 13.0023, 13.0024				5/5
O123/O186		O123 std., 8.0269, 7.0571 5.0769, 15.0862				5/5
O128		O128 std., 13.0062, 13.0027, 13.1024, 14.0696				5/5

O138		O138 std., 13.1113	2/2
O171	1044-1, 12772		2/2
O175		O175 std., 9.1703, 10.0047, 10.0048, 10.0472	5/5

Table 2C. Validation of multiplex PCR assay set no. 3 to detect 'non-top-7' Shiga toxin producing *Escherichia coli* serogroups

Serogroup	Source of strains, Strain IDs					No. of strains positive/No. of strains tested
	Kansas State University	Pennsylvania State University	Michigan State University	University of Nebraska	Food and Drug Administration	
O6	5750-1, 16857, 8276-1					3/3
O25		O25 std., 3.1421 14.1138, 14.1119, 14.0200, 14.0052				6/6
O33		O33 std., 13.0050, 13.1199, 15.1254, 16.0504				5/5
O75		O75 std., 11.1836, 12.0940, 12.0944, 12.1624				5/5
O79		O79 std., 5.2457				2/2
O98	sPRH-571	13.1267, 15.0710				3/3

O116	1238-1, 1582-1, 99-3708, 3702-1, 7712-1, 7716, 7752-2		7/7
O150		12.3283, 12.3280, 12.3284, 3.4896, 3.4897	5/5
O181		O181 std., 11.0228, 11.0392, 12.0697	4/4

Table 2D. Validation of multiplex PCR assay set no. 4 to detect 'non-top-7' Shiga toxin producing *Escherichia coli* serogroups

Serogroups	Source of strains, Strain IDs					No. of strains positive/No. of strains tested
	Kansas State University	Pennsylvania State University	Michigan State University	University of Nebraska	Food and Drug Administration	
O2/O50	99-18866, 99-18858	14.1652				3/3
O15	2794, 18925-1, S918, s915, N5789, N15018	O13 std.	TW1681			8/8
O76		O76 std., 14.0706, 14.0760, 14.0940, 14.0986				5/5
O78	ATCC 35401, ATCC 43896	O78 std., 10.0182				4/4
O113	1598-2, 1862-1, 3517					3/3
O118/O151	KDHE 14, KDHE 52	13.1018				3/3
O126	99-17409	16.0457, 16.0511, 13.0069				4/4
O146						5/5

		7.2907, 7.2663, 7.2803, 7.3159, 14.0976		
O147	13466-1		TW7464	2/2
O178		O178 std., 14.0800, 14.0828, 14.0829, 14.0908		5/5

Table 2E. Validation of multiplex PCR assay set no. 5 to detect 'non-top-7' Shiga toxin producing *Escherichia coli* serogroups

Serogroups	Source of strains, Strain IDs					No. of strains positive/No. of strains tested
	Kansas State University	Pennsylvania State University	Michigan State University	University of Nebraska	Food and Drug Administration	
O7		16.0405, 14.0598, 14.0551				3/3
O8	2089-2, 4202, 4950-2, sPRH-559, 5702-1, 6102-2, 6504-1, 6846-2, 6849-1, 15484-1,4544-1, 4964-1, 18370-1, 4542-1, 6412-1, 6416-1, 5344-1, 5799	86.1006				19/19
O20	5481					1/1
O55	5906, ATCC 12014		TW04062, TW08260			4/4
O62/O68		O68 std., 1.2557, 4.0175, 4.2378				4/4

O87		15.0108,<br>14.0979,<br>12.3233	3/3
O92		13.0124	1/1
O136	4709-1,<br>10314-1,<br>10320-1	14.0889	4/4
O163	9388-1,<br>13802-1,<br>18917-1		3/3

Table 2F. Validation of multiplex PCR assay set no. 6 to detect 'non-top-7' Shiga toxin producing *Escherichia coli* serogroups

Serogroups	Source of strains, Strain IDs					No. of strains positive/No. of strains tested
	Kansas State University	Pennsylvania State University	Michigan State University	University of Nebraska	Food and Drug Administration	
O38	748-1, 13950-1, 2072-3					3/3
O39		13.1182, 15.1383, 16.1097				3/3
O74	4558-1, 8608 13472-1, 1229-1					4/4
O88	1235-1, 2076-2, 8652-1, 4560-1, 1240-2					5/5
O96	497, 3712-1, 18862-1, 3714-1					4/4
O107/O117	3536-3, 9966-1 3534-1					3/3
O108	9924-1					1/1

O115		13.1261	1/1
O130	492-1,1038-2,<br>1239-2,<br>3270-1		4/4
O132	2067-1,<br>sPRH-568		2/2
O141		13.1660,<br>14.0312<br>14.0314	3/3
O153	1932, 9916-1		2/2

Table 2G. Validation of multiplex PCR assay set no. 7 to detect 'non-top-7' Shiga toxin producing *Escherichia coli* serogroups

Serogroups	Source of strains, Strain IDs					No. of strains positive/No. of strains tested
	Kansas State University	Pennsylvania State University	Michigan State University	University of Nebraska	Food and Drug Administration	
O1		14.0223			6227	2/2
O17/O44/O73/O77/O106		8.2525, 8.2526 14.0994, 14.0989, 14.0398			3829	6/6
O18		12.0213, 13.0442, 13.1139			6853	4/4
O28		4.0349, 5.0190			6404-2	3/3
O35		1.2208, 6.089			6709	3/3
O37		5.0628, 7.3209			6594	3/3
O40		12.0572, 13.0486				2/2
O43		12.0257, 15.1752, 16.0015			6861	4/4

O51	11.1861,<br>12.2022,<br>15.0773	3724	4/4
O53	12.0583	6503	2/2
O69	14.1063,<br>14.1064	3560	3/3
O70	95.1266,<br>96.1774	6388	3/3

Table 2H. Validation of multiplex PCR assay set no. 8 to detect 'non-top-7' Shiga toxin producing *Escherichia coli* serogroups

Serogroups	Source of strains, Strain IDs					No. of strains positive/No. of strains tested
	Kansas State University	Pennsylvania State University	Michigan State University	University of Nebraska	Food and Drug Administration	
O81		14.1380, 14.1397			6754	3/3
O82		14.0846, 15.0429			5541	3/3
O85		15.1549, 16.0447			3620	3/3
O90/O127		12.3598	TW4549			2/2
O102		12.3542, 12.3624, 14.0030			3558	4/4
O105		9.0695, 9.0698			6810	3/3
O124/O164		15.0880, 15.0790				2/2
O125		13.0499, 13.1058			6517	3/3
O139		13.0785, 14.0081			6672	3/3
O140		12.066			5737	2/2

O148		12.3461,<br>13.1357	2/2

Table 2I. Validation of multiplex PCR assay set no. 9 to detect 'non-top-7' Shiga toxin producing *Escherichia coli* serogroups

Serogroups	Source of strains, Strain IDs					No. of strains positive/No. of strains tested
	Kansas State University	Pennsylvania State University	Michigan State University	University of Nebraska	Food and Drug Administration	
O21		9.0069, 12.3518			6508	3/3
O49		13.0005			5598	2/2
O93		87.1672			6613	2/2
O110		12.2044			3744	2/2
O114		12.0637, 13.1247			6583	3/3
O149		12.2184, 13.1187			6495	3/3
O154		13.0130, 14.0265			6742	3/3
O161					6341	1/1
O169		12.0689			6992	2/2

Table 2J. Validation of multiplex PCR assay set no. 10 to detect 'non-top-7' Shiga toxin producing *Escherichia coli* serogroups

Serogroups	Source of strains, Strain IDs					No. of strains positive/No. of strains tested
	Kansas State University	Pennsylvania State University	Michigan State University	University of Nebraska	Food and Drug Administration	
O46/O134	1223-4	12.1020, 15.0242, 16.0631			3606	5/5
O152		4.2414, 7.0560				2/2
O159	1554-1	12.1465				2/2
O160		15.1575, 14.0525				2/2
O165	7050-4	13.0107, 14.1095			6757	4/4
O170		13.0772, 14.1230			6833	3/3
O172	9042-1, 11154-1					2/2
O174		14.153			6129	2/2
O176		14.0193			6996	2/2

O177		14.1088, 14.1094	E28F-5, 89H-4, 58H-1		5/5
O179		12.0472, 12.0696, 11.1855, 11.1606			4/4
O182	sPRH-569	13.151, 14.1296		5745	4/4

Table 2K. Validation of multiplex PCR assay set no. 11 to detect 'non-top-7' Shiga toxin producing *Escherichia coli* serogroups

Serogroups	Source of strains, Strain IDs					No. of strains positive/No. of strains tested
	Kansas State University	Pennsylvania State University	Michigan State University	University of Nebraska	Food and Drug Administration	
O3		14.1045, 14.1048			6291	3/3
O10		7.1714			6888	2/2
O11		13.0841, 13.0861			6812	3/3
O16					6734	1/1
O19		14.0988, 15.0093, 16.054				3/3
O23		1.2315, 3.2841			6556	3/3
O29	10720-1	1.1116			3608	3/3
O63		3.2810, 3.4555			6441	3/3
O101/O162				E907F-2		1/1
O112		14.1406, 14.1409			5127	3/3
O131		12.3205, 13.0803			6901	3/3

Table 2L Validation of multiplex PCR assay set no. 12 to detect 'non-top-7' Shiga toxin producing *Escherichia coli* serogroups

Serogroups	Source of strains, Strain IDs					No. of strains positive/No. of strains tested
	Kansas State University	Pennsylvania State University	Michigan State University	University of Nebraska	Food and Drug Administration	
O9					6420, 6625	2/2
O27					6683	1/1
O41					6484, 6923	2/2
O48					6134, 6589	2/2
O54					6285, 6316	2/2
O56					5184	1/1
O60					3549, 5007	2/2
O142					5310, 6469	2/2
O143					5472, 6091	2/2

Table 2M. Validation of multiplex PCR assay set no. 13 to detect 'non-top-7' Shiga toxin producing *Escherichia coli* serogroups

Serogroup	Source of strains, Strain IDs					No. of strains positive/No. of strains tested
	Kansas State University	Pennsylvania State University	Michigan State University	University of Nebraska	Food and Drug Administration	
O12					5736, 6756	2/2
O58					5900, 6698	2/2
O64					6400, 6661	2/2
O83					6046, 6431	2/2
O133					5695	1/1
O166					6593, 6649	2/2
O167					6743	1/1

Table 2N. Validation of multiplex PCR assay set no. 14 to detect 'non-top-7' Shiga toxin producing *Escherichia coli* serogroups

Serogroups	Source of strains, Strain IDs					No. of strains positive/No. of strains tested
	Kansas State University	Pennsylvania State University	Michigan State University	University of Nebraska	Food and Drug Administration	
O32					5773, 6555, 6573	3/3
O65					5108, 5952	2/2
O66					6598, 6918	2/2
O71					3565, 6829	2/2
O100					6187, 6395	2/2
O144					OR1	1/1
O173					5984	1/1
O180					5026, 6270	2/2
