# Supplementary material for: Multiplex PCR Assays for the Detection of One Hundred and Thirty Seven Serogroups of Shiga Toxin-Producing Escherichia coli Associated With Cattle
Source: Front Cell Infect Microbiol. 2020 Jul 29;10:378. doi: 10.3389/fcimb.2020.00378 (PMC7403468; doi:10.3389/fcimb.2020.00378)

QIAxcel images of the 14 sets of multiplex PCR assays targeting 145 serogroups of Shiga toxin-producing *Escherichia coli* for testing specificity of each assay set. Each assay was run with DNA from pooled cultures of the targeted serogroup (PC), DNA from pooled cultures for the other 13 sets and top-7 plus O104, and negative control (NC), which included all reagents except the DNA template.

mPCR assay set 1

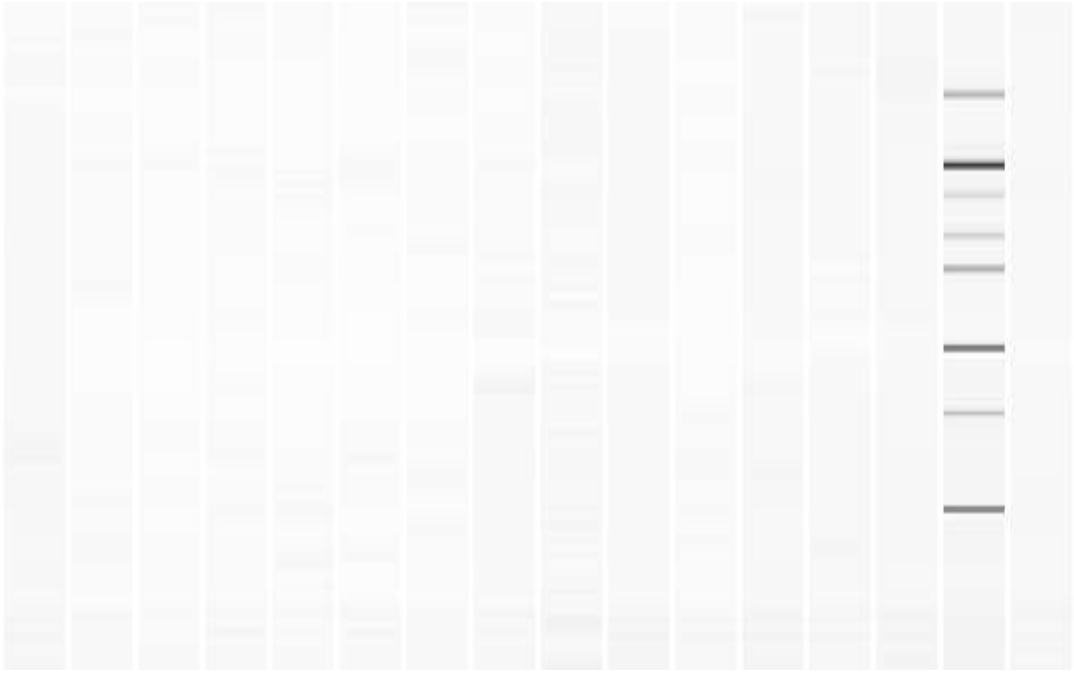

Set 2 | Set 3 | Set 4 | Set 5 | Set 6 | Set 7 | Set 8 | Set 9 | Set 10 | Set 11 | Set 12 | Set 13 | Set 14 | Top-7 +104 | PC | NC

O group	bp
O4	832
O86	562
O84	501
O156	452
O80	406
O168	336
O91	277
O109	204

mPCR assay set 2

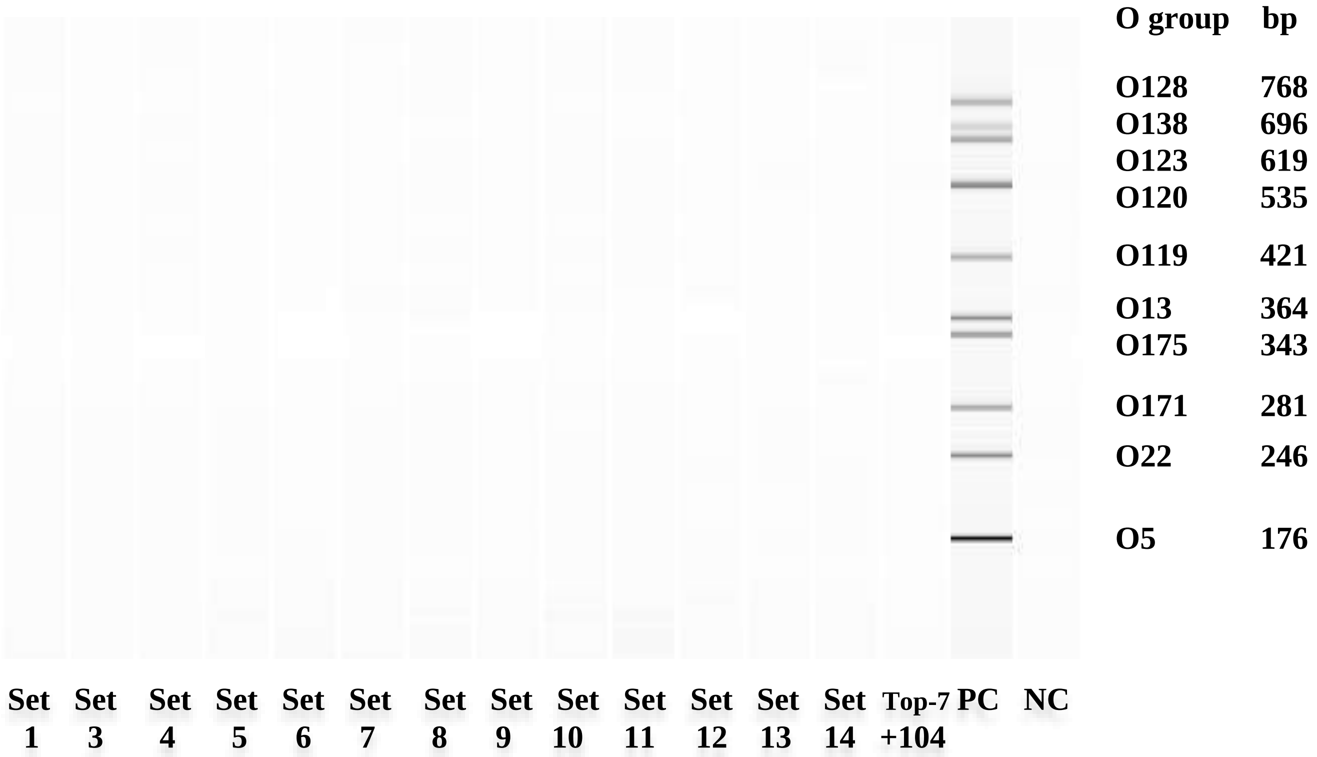

mPCR assay set 3

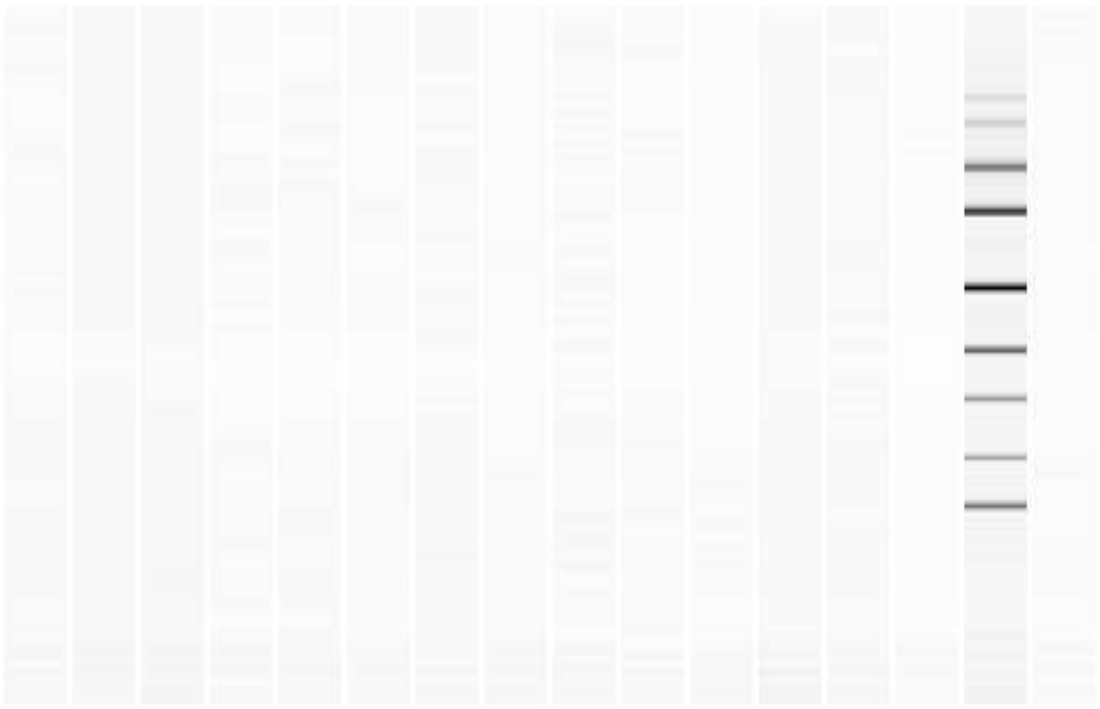

O group	bp
O6	783
O98	675
O181	595
O75	511
O33	413
O116	355
O150	313
O79	266
O25	230

mPCR assay set 4

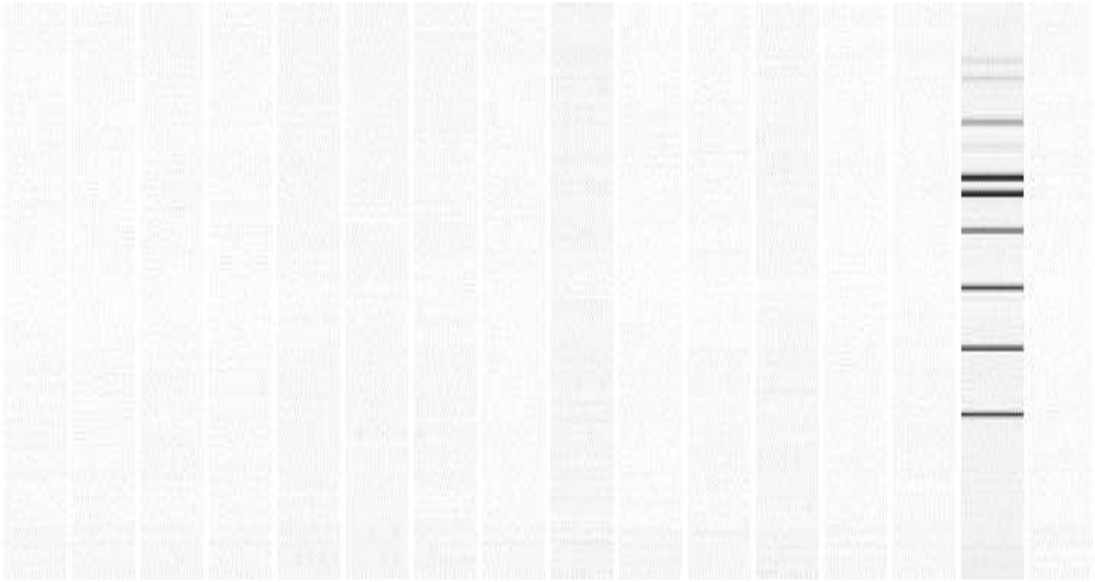

Set 1 | Set 2 | Set 3 | Set 5 | Set 6 | Set 7 | Set 8 | Set 9 | Set 10 | Set 11 | Set 12 | Set 13 | Set 14 | Top-7 +O104 | PC | NC

O group	bp
O78	992
O2	819
O146	640
O76	533
O178	495
O126	465
O113	419
O118	344
O15	288
O147	230

mpCR assay set 5

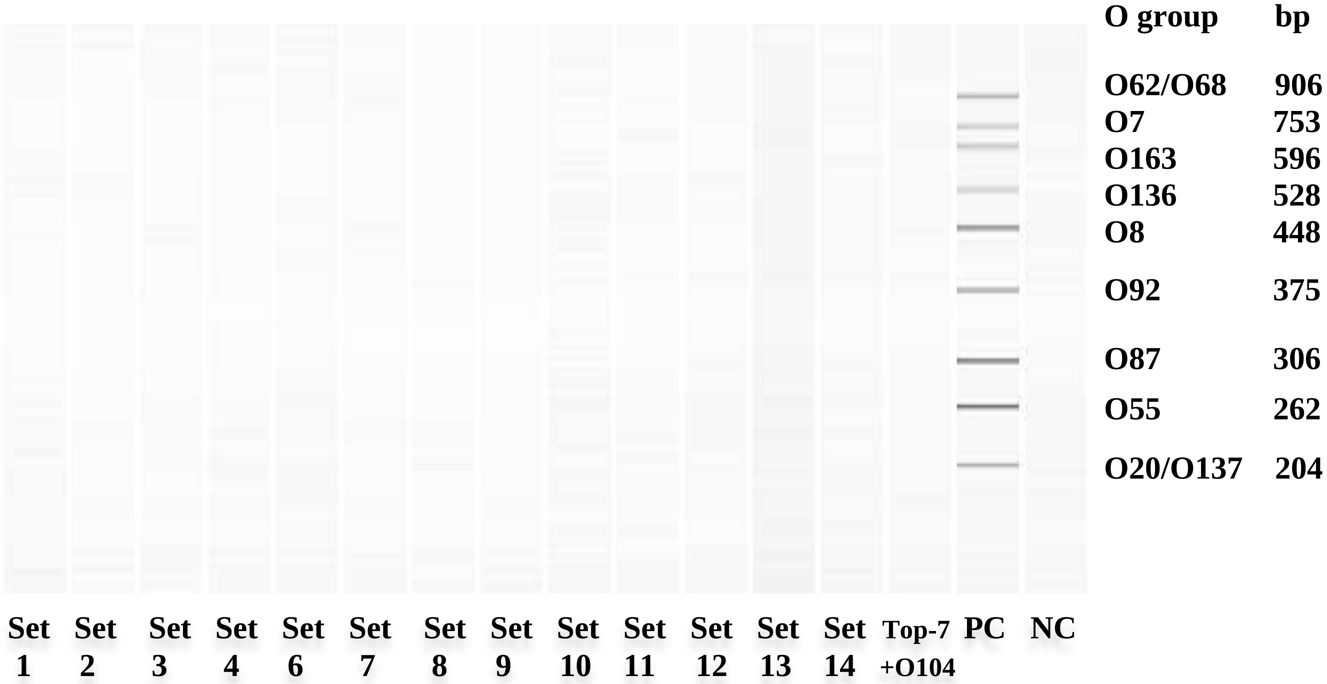

mPCR assay set 6

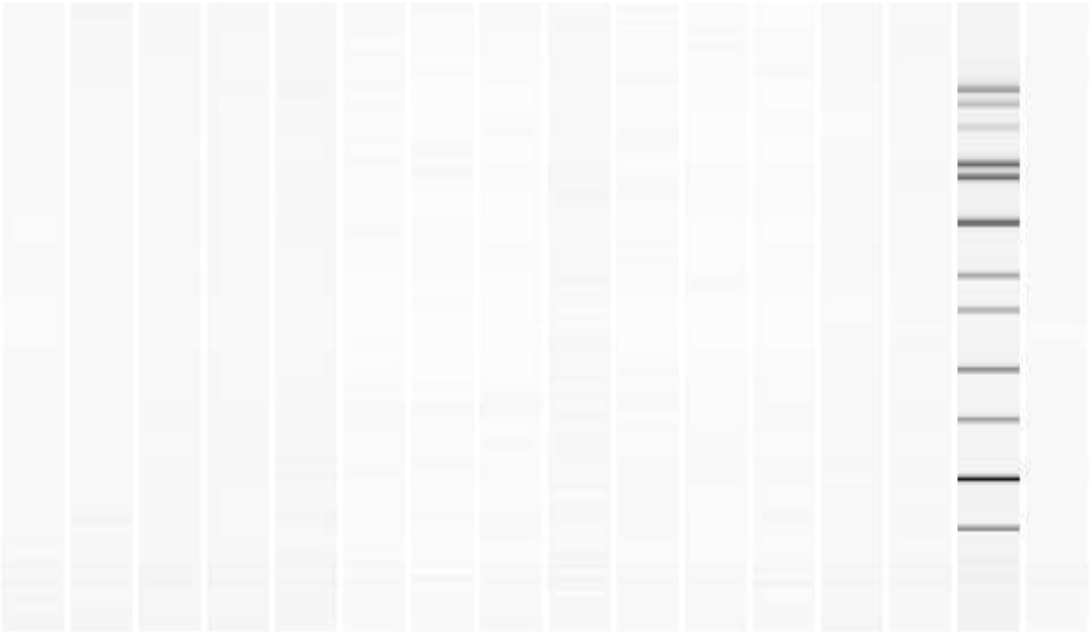

Set 1 | Set 2 | Set 3 | Set 4 | Set 5 | Set 7 | Set 8 | Set 9 | Set 10 | Set 11 | Set 12 | Set 13 | Set 14 | Top-7 +O104 | PC | NC

O group	bp
O141	880
O153	741
O132	652
O130	567
O108	515
O96	457
O88	394
O107/117	357
O74	303
O38	253
O39	201
O115	158

mPCR assay set 7

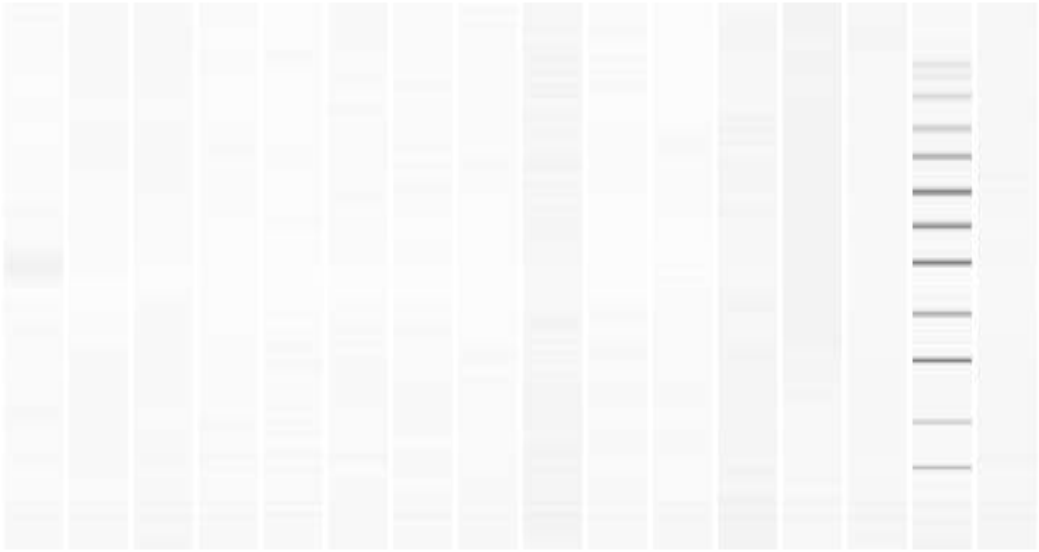

Set 1 | Set 2 | Set 3 | Set 4 | Set 5 | Set 6 | Set 8 | Set 9 | Set 10 | Set 11 | Set 12 | Set 13 | Set 14 | Top-7 +O104 | PC | NC

O group	bp
O70	863
O53	735
O69	649
O51	566
O17/44/73/77/106	500
O43	445
O40	396
O37	353
O35	305
O28 (O42/O20ac)	255
O18 (18ab/18ac)	199
O1	152

mPCR assay set 8

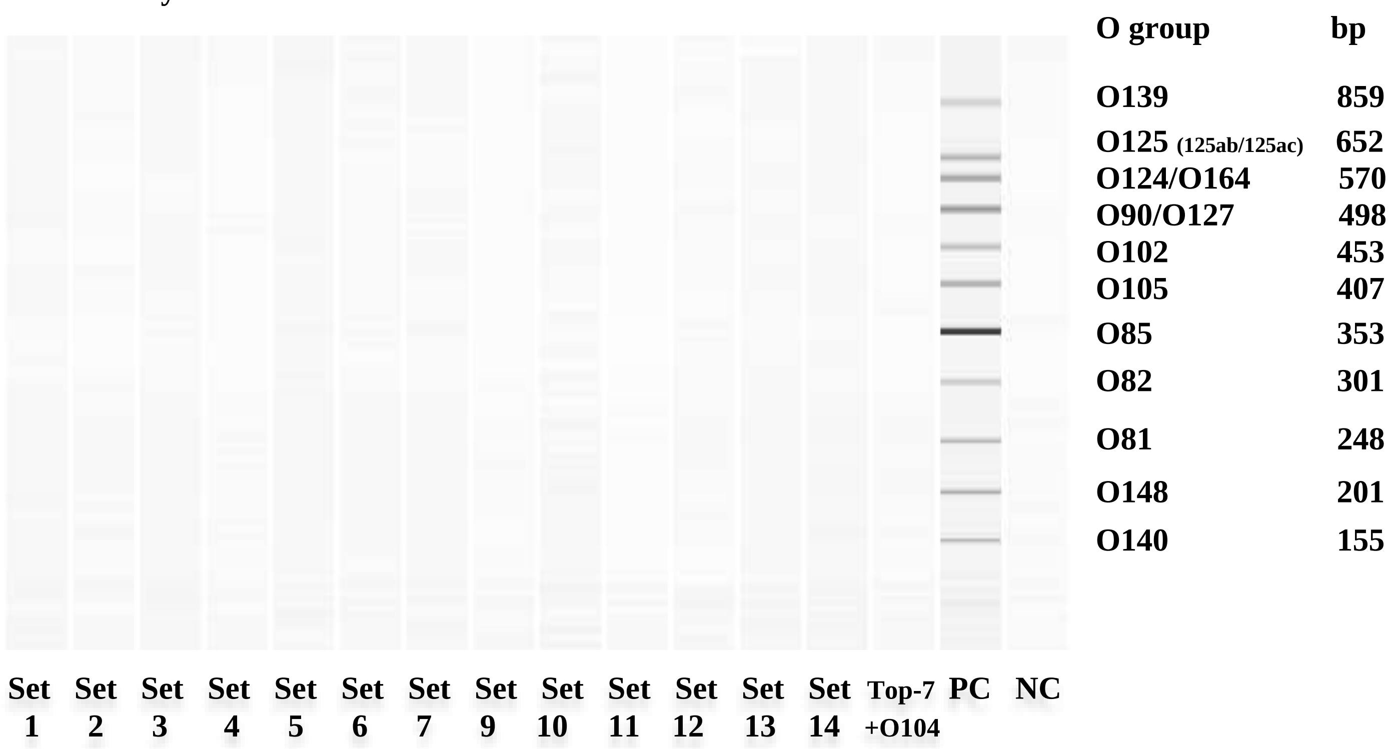

mPCR assay set 9

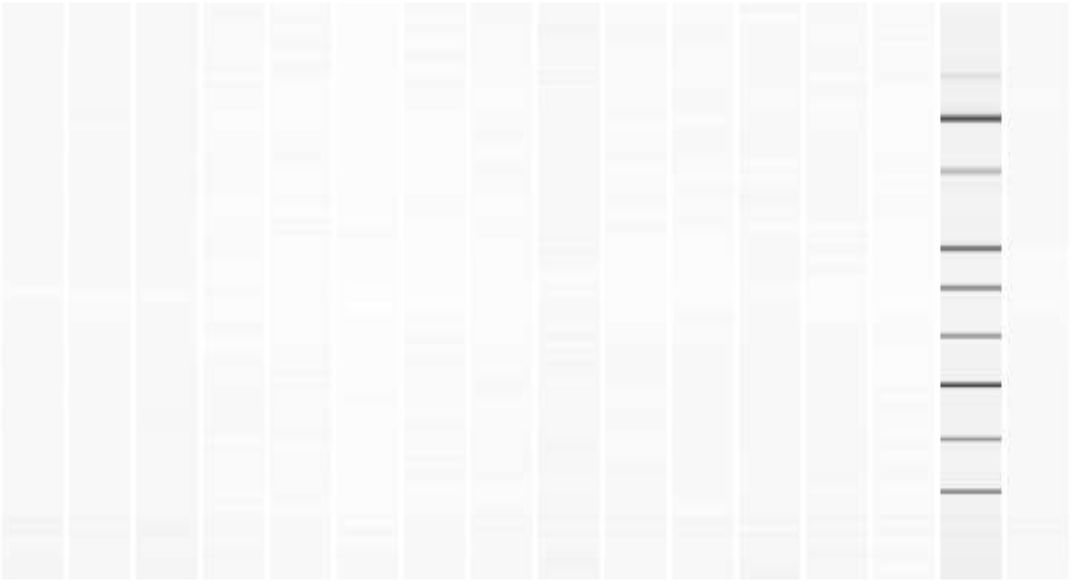

mPCR assay set 10

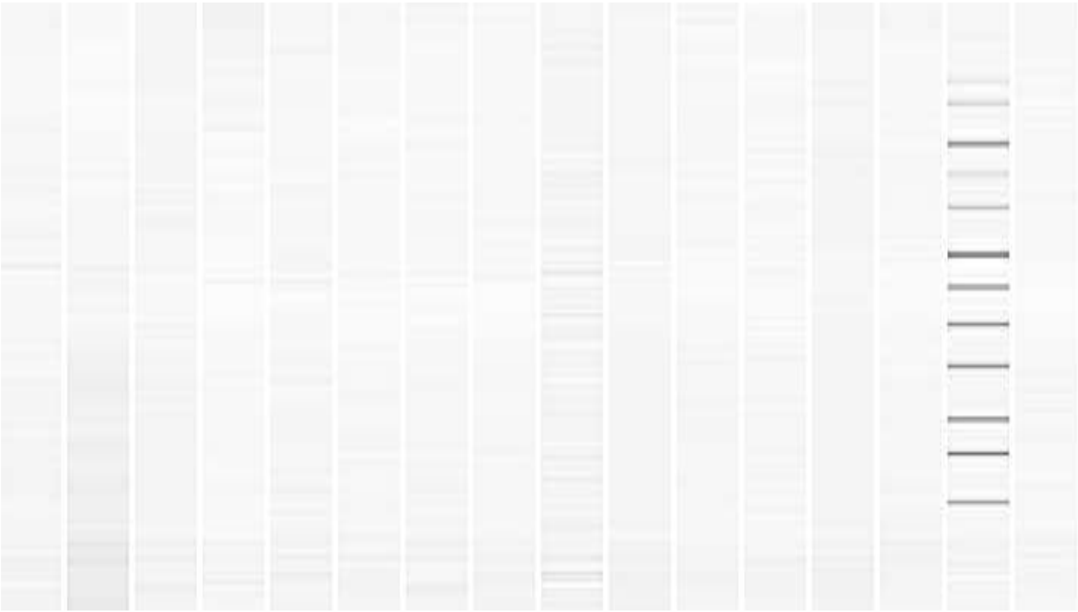

O group	bp
O165	735
O160	655
O182	566
O179	505
O46/O134	455
O177	395
O176	356
O174	317
O172	278
O170	233
O159	202
O152	150

Set 1 Set 2 Set 3 Set 4 Set 5 Set 6 Set 7 Set 8 Set 9 Set 11 Set 12 Set 13 Set 14 Top-7 +O104 PC NC

mPCR assay set 11

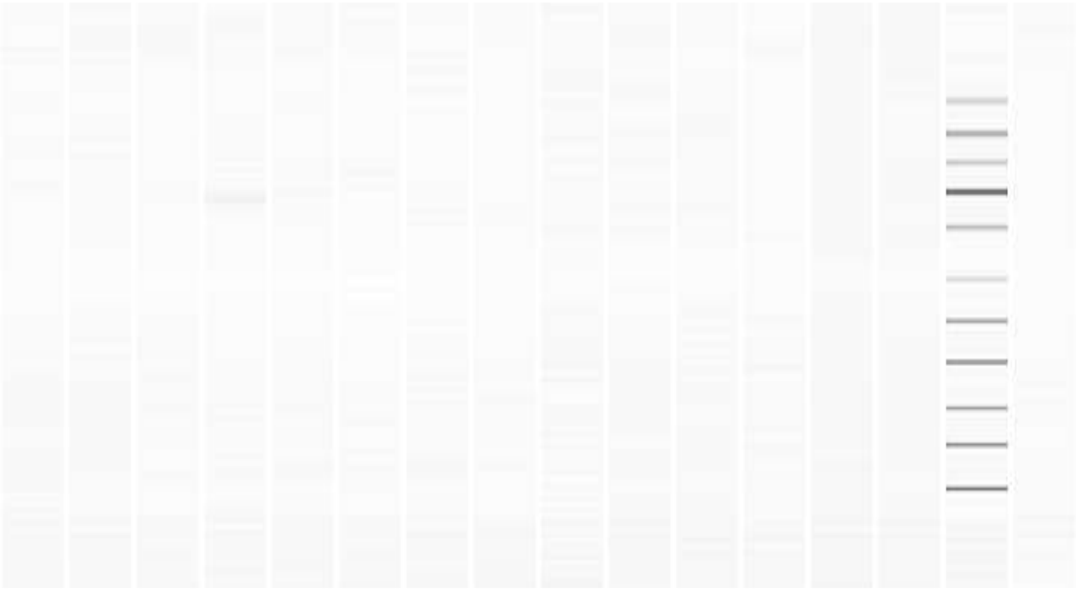

O group	bp
O131	655
O19	574
O16	505
O63	455
O23	403
O29	348
O101/O162	309
O112ab	270
O11	225
O10	187
O3	145

Set 1 Set 2 Set 3 Set 4 Set 5 Set 6 Set 7 Set 8 Set 9 Set 10 Set 12 Set 13 Set 14 Top-7 +O104 PC NC

mPCR assay set 12

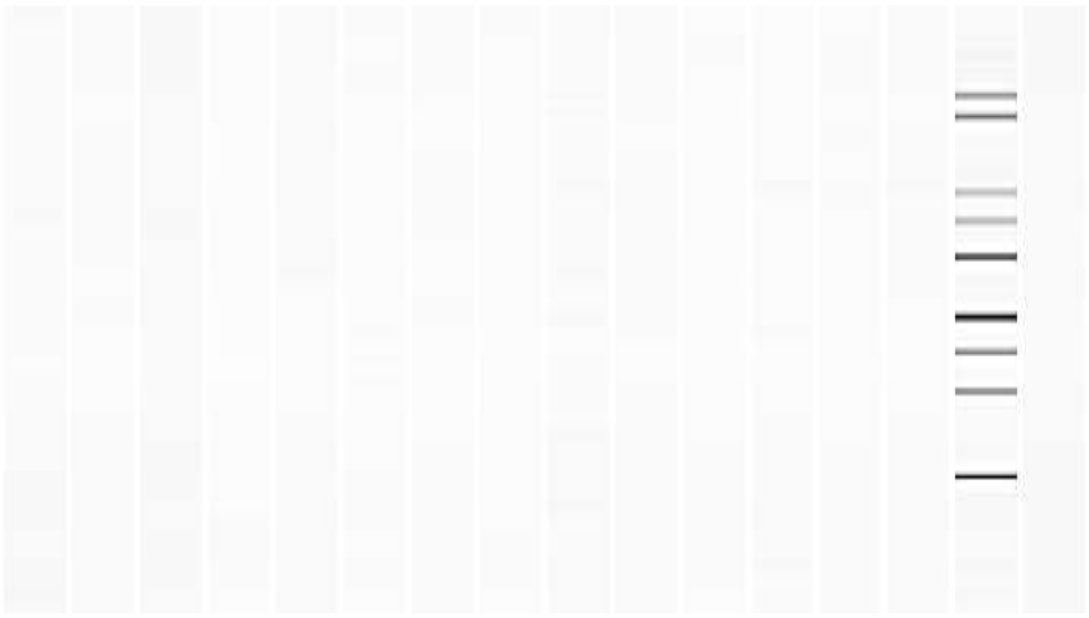

mPCR assay set 13

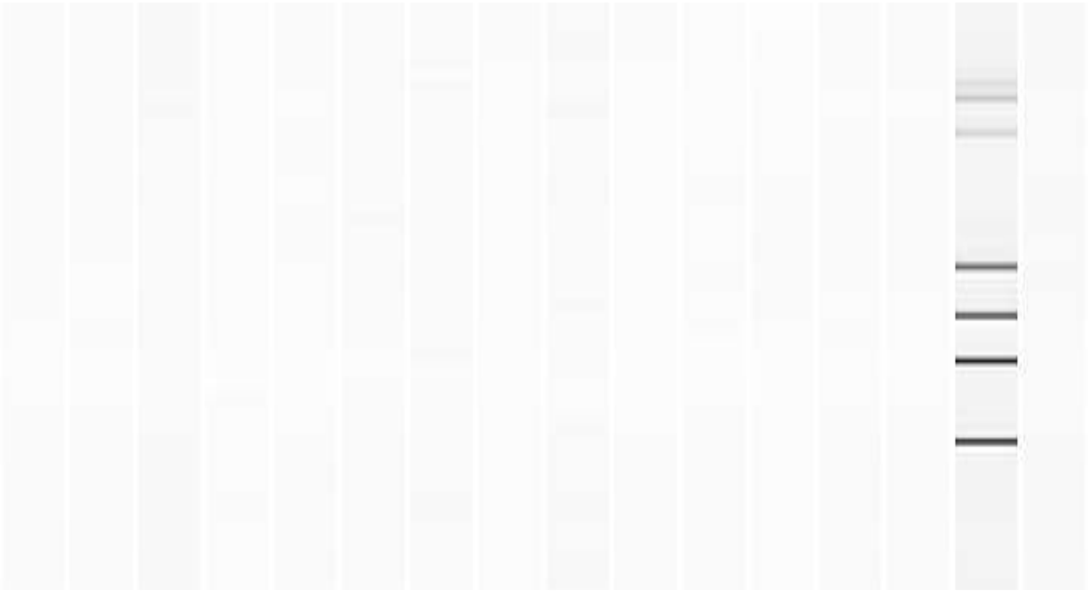

O group	bp
O58	1046
O12	885
O64	727
O166	462
O167	403
O83	362
O133	294

Set 1 Set 2 Set 3 Set 4 Set 5 Set 6 Set 7 Set 8 Set 9 Set 10 Set 11 Set 12 Set 14 Top-7 +O104 PC NC

mPCR assay set 14

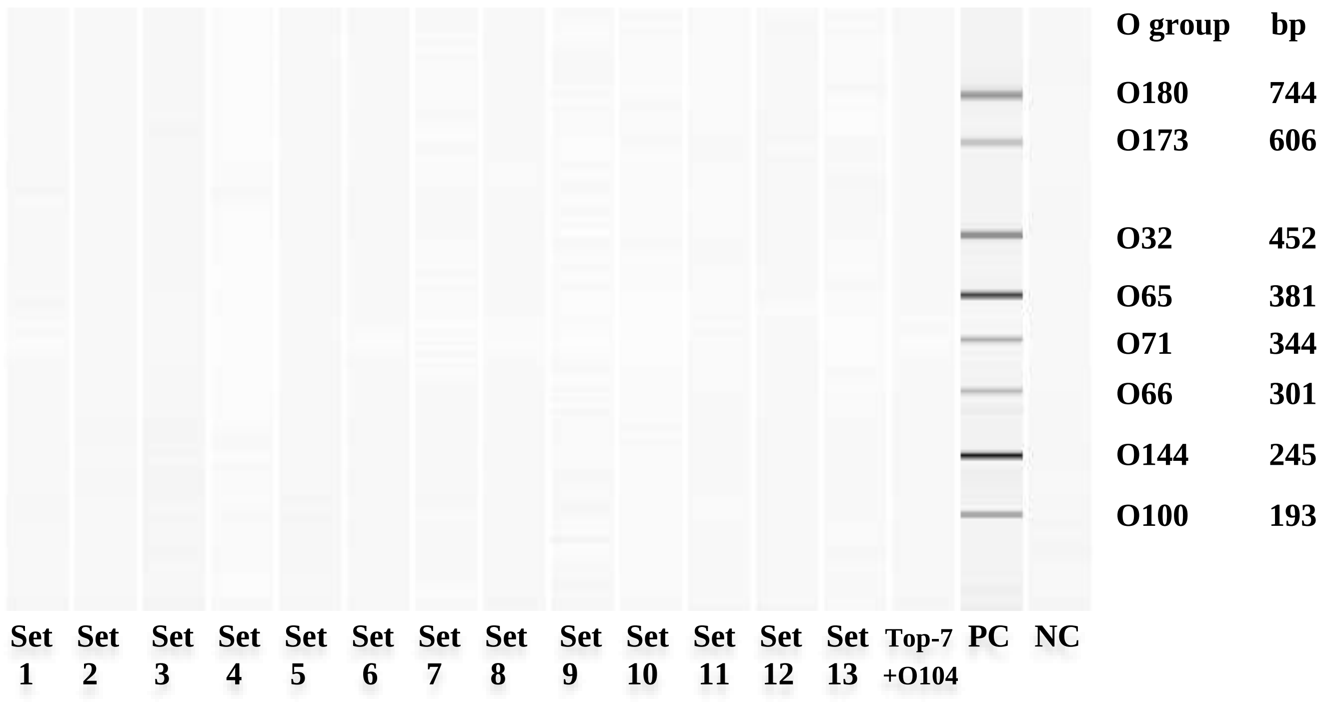

Supplement: Supplementary file 3 [file Data_Sheet_3.pdf]
